# CTCF and cohesin promote focal detachment of DNA from the nuclear lamina

**DOI:** 10.1186/s13059-022-02754-3

**Published:** 2022-09-01

**Authors:** Tom van Schaik, Ning Qing Liu, Stefano G. Manzo, Daan Peric-Hupkes, Elzo de Wit, Bas van Steensel

**Affiliations:** 1 Oncode Institute and Division of Gene Regulation, Netherlands Cancer Institute, Amsterdam, the Netherlands; 2 Present address: Annogen, Amsterdam, the Netherlands; 3 Department of Cell Biology, Erasmus University Medical Center, Rotterdam, the Netherlands

**Keywords:** Nuclear lamina, Lamina-associated domains, pA-DamID, CTCF, Cohesin, Acute protein depletion, Local detachment, Heterochromatin

## Abstract

**Background:**

Lamina-associated domains (LADs) are large genomic regions that are positioned at the nuclear lamina. It has remained largely unclear what drives the positioning and demarcation of LADs. Because the insulator protein CTCF is enriched at LAD borders, it was postulated that CTCF binding could position some LAD boundaries, possibly through its function in stalling cohesin and hence preventing cohesin invading into the LAD. To test this, we mapped genome–nuclear lamina interactions in mouse embryonic stem cells after rapid depletion of CTCF and other perturbations of cohesin dynamics.

**Results:**

CTCF and cohesin contribute to a sharp transition in lamina interactions at LAD borders, while LADs are maintained after depletion of these proteins, also at borders marked by CTCF. CTCF and cohesin may thus reinforce LAD borders, but do not position these. CTCF binding sites within LADs are locally detached from the lamina and enriched for accessible DNA and active histone modifications. Remarkably, despite lamina positioning being strongly correlated with genome inactivity, this DNA remains accessible after the local detachment is lost following CTCF depletion. At a chromosomal scale, cohesin depletion and cohesin stabilization by depletion of the unloading factor WAPL quantitatively affect lamina interactions, indicative of perturbed chromosomal positioning in the nucleus. Finally, while H3K27me3 is locally enriched at CTCF-marked LAD borders, we find no evidence for an interplay between CTCF and H3K27me3 on lamina interactions.

**Conclusions:**

These findings illustrate that CTCF and cohesin are not primary determinants of LAD patterns. Rather, these proteins locally modulate NL interactions.

**Supplementary Information:**

The online version contains supplementary material available at 10.1186/s13059-022-02754-3.

## Background

The nuclear lamina (NL) is positioned near the inner nuclear membrane and consists of a protein meshwork of A- and B-type lamins and a variety of other proteins. Peripherally positioned chromatin interacts with the NL and comprises approximately a thousand large genomic domains that are up to 10 Mb in size [1]. These lamina-associated domains (LADs) are strongly depleted for active genes and are thought to provide a backbone for genome organization. LADs are highly conserved between cell types and species, but the mechanisms that underlie genome positioning at the NL remain poorly understood (reviewed in [2–4]).

LADs are strongly enriched for features of heterochromatin. Besides having a low gene density and typically low gene expression levels, LADs replicate late in S-phase, are visible by electron microscopy as densely packed chromatin, and are enriched for the histone modifications H3K9me2 and H3K9me3 [1, 5–9]. These heterochromatin characteristics are important because gene activation, chromatin decondensation, and H3K9me2/3 perturbation all result in a loss of DNA–NL interactions [7, 10–14]. LAD interactions with the NL are mediated by various proteins in a redundant manner, including but probably not limited to Lamin B receptor (LBR), Lamin A, and CEC-4 (the latter only in *C. elegans*) [13, 15]. Differences in NL interactions between cell types are likely caused by variable epigenetic landscapes and protein compositions of the NL.

One particular aspect of LADs that remains poorly understood is the sharp transition in NL interactions at LAD borders, which suggests the existence of specific mechanisms to define these borders. LAD borders are enriched for active promoters, CpG islands (often at active promoters), and binding of the insulator protein CTCF [1]. While there is increasing evidence that gene activity results in local detachment from the NL [11, 14], which could explain a sharp transition at some borders, the role of CTCF on LAD border positioning is still unclear. Because few borders are marked by both CTCF and active promoters, it was postulated that CTCF may independently demarcate at least a subset of LAD borders [1, 12]. In support of this hypothesis, a deletion of a ~30-kb LAD border region at the *Tcrb* locus resulted in decreased NL interactions up to 100 kb inside the LAD, and this behavior was almost completely recapitulated by deletion of a ~4.5-kb region that included all three CTCF binding sites at this border [16]. However, a genome-wide view of a possible causal role of CTCF in LAD border formation is still lacking.

CTCF is a broadly expressed and essential zinc-finger protein that interacts with the ring-shaped cohesin complex on the genome [17, 18]. Once loaded on DNA, cohesin is thought to establish long-range DNA contacts through a processive increase in chromatin loop size, a process known as extrusion ( [19, 20], reviewed in [21]). Extrusion is halted by the interaction of the SA1/2-RAD21 interface of the cohesin complex with the N-terminal regions of CTCF [22, 23]. Cohesin is actively loaded by the NIPBL/MAU2 complex and unloaded by the cohesin release factor WAPL to maintain a continuous cycle of loop extrusion [24, 25]. Together with other factors, such as cell-type-specific transcription factors, cohesin mediates a continuous cycle of loop extrusion to preserve distal gene regulation [26]. Remarkably, besides having extended DNA loops, WAPL-knockout cells also have somewhat fragmented LADs [25]. However, the mechanism by which WAPL loss affects NL interactions remains to be elucidated.

In addition to the genomic features discussed above, in some cell types, H3K27me3 is also enriched near LAD borders [1, 12]. H3K27me3 and CTCF could be involved in LAD demarcation together, given that both were reported to be involved in the peripheral positioning of ectopically integrated LAD fragments [12]. However, the role of H3K27me3 may be cell-type specific, as this mark is not correlated with NL interactions in all cell types [10, 27]. Additionally, H3K27me3 is associated with dynamic LADs during the cell cycle [28], oncogene-induced senescence [29], and strain-induced genome repositioning [30].

Two recent developments now allowed us to investigate the interplay between LAD borders and CTCF and cohesin. First, the fusion of endogenous proteins with auxin-inducible degron (AID) tags mediates rapid and near-complete protein depletion, which is particularly useful for essential proteins or complexes such as CTCF and cohesin [31, 32]. Second, the application of proteinA-DamID (pA-DamID) results in the mapping of genome–NL interactions with a high temporal resolution, which is required to study the effects of rapid protein depletion [33]. Here, using a combination of these two tools in mouse embryonic stem cells (mESCs), we show that CTCF and cohesin mediate focal detachment from the NL, but have limited effects on LAD border positioning. On a chromosomal scale, perturbation of cohesin dynamics induces quantitative changes of NL interactions, which signifies that cohesin affects the radial positioning of the genome. Finally, pharmacological depletion of H3K27me3 does not affect NL interactions in mESCs, even in combination with CTCF depletion.

## Results

### LAD borders are enriched for CTCF binding in multiple cell lines

**Fig. 1:**
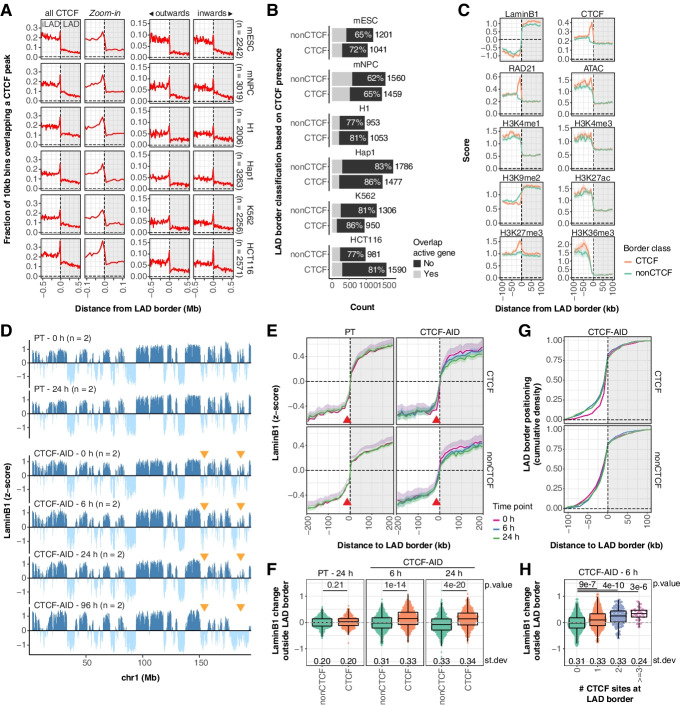
NL interactions are largely maintained upon CTCF depletion. **A** Positioning of CTCF binding sites around LAD borders in various mouse and human cell lines. *n* shows the number of LAD borders for every cell type (see the “Methods” section for more details). Data are from refs [25, 26, 33–35]. **B** LAD borders are classified as CTCF borders if a CTCF site is positioned within 20 kb outside of the LAD (overlapping with the enriched CTCF density). The percentage of LAD borders that is not within 10 kb of active genes (FPKM > 1) is highlighted. Data are from refs [25, 26, 35–39]. **C** Average scores for LaminB1 DamID (log_2_ ratio over Dam), CTCF and cohesin binding (ChIP-seq coverage), and various publicly available epigenetic data sets around mESC LAD borders, classified by CTCF presence. Solid lines and shaded areas represent the mean signals and 95% confidence intervals of the mean, respectively. Data are from [26, 40–44]. **D** LaminB1 pA-DamID *z*-scores along a representative chromosome for mESC parental (PT) and CTCF-AID cells, for a time course of IAA addition in (0, 6, 24, and 96 h). Data tracks are processed in 10-kb bins and averages of *n* biological replicates. Orange arrows highlight example regions with variable signals following CTCF depletion. **E** Average LaminB1 *z*-scores (except 96 h) around LAD borders (from mESC PT pA-DamID data, Additional file 1: Fig. S2E). The solid line and the shaded area represent the mean signal and 95% confidence interval of the mean, respectively. The red arrow highlights the position of the CTCF enrichment. **F** Quantification of the LaminB1 changes with 0 h at the red arrow in **E** for all individual LAD borders. A Wilcoxon test was used to test for statistical significance between borders with and without CTCF, followed by Benjamini–Hochberg multiple testing correction. **G** Cumulative density profile of LAD border positioning as determined by hidden Markov modeling of the CTCF-AID samples, relative to the nearest LAD border from the PT clone. A distance cutoff of 100 kb was used to prevent comparisons between different LADs. **H** Similar plot to **F** for CTCF treated with 6 h of IAA, but further segmented by the number of CTCF sites at the LAD border

We first set out to explore the correlation between CTCF binding (as detected by ChIP-seq) and LAD borders in more detail by comparing these in two mouse and four human cell lines with available NL interaction maps (obtained by DamID). CTCF binding is consistently depleted within LADs and locally enriched near LAD borders, peaking 10 kb outside LADs (Fig. 1A). CTCF is enriched at cell-type-specific borders and at LAD borders shared between cell types (Additional file 1: Fig. S1A), although shared borders often have overall slightly elevated CTCF density. Thus, CTCF enrichment at LAD borders is not specific to either stable or variable borders.

CTCF binds the genome in an oriented manner due to a non-palindromic motif. Loops primarily occur between two convergent CTCF motifs [45–47]. To test whether LAD borders are enriched for a certain orientation, directionality of CTCF binding sites was assigned based on the orientation of its motif. We observe that LAD borders are enriched for CTCF binding sites with both motif orientations (Fig. 1A) and therefore are likely involved in DNA looping towards (*inwards*) and away from (*outwards*) LADs.

CTCF is thus positioned at a subset of LAD borders, which raises the question whether these borders are different from LAD borders without CTCF. We classified all LAD borders into two categories based on nearby CTCF binding (positioned within 20 kb outside borders, overlapping with the observed enrichment), which results in 42–62% being classified as “CTCF borders” for the different cell types (Fig. 1B). In accordance with previous observations [1], LAD borders are also enriched for transcription, indicated by a local enrichment of transcription start sites (TSS) and transcription end sites (TES) for genes transcribing from and towards the LAD, respectively (Additional file 1: Fig. S1B). Active genes are present at LAD borders with and without CTCF binding (Fig. 1B). Because transcription is known to induce NL detachment locally [14], we excluded borders near active genes from further analyses unless otherwise indicated, to avoid confounding effects from transcription on NL interactions and LAD border positioning.

Average profiles of NL interactions, CTCF, and cohesin binding and publicly available epigenetic data [26, 40–44] at mESC LAD borders reveal that borders with CTCF generally show a somewhat sharper transition between LAD and inter-LAD (iLAD), for example in NL interactions, ATAC-seq and H3K4me1 (Fig. 1C). As expected, LAD borders with CTCF binding are locally enriched for RAD21 binding (a subunit of the cohesin complex). Finally, LAD borders with CTCF binding are locally enriched for H3K27me3 and depleted for H3K9me2. All of these enrichments are irrespective of the orientation of the CTCF motif (Additional file 1: Fig. S1C-D) and are mostly conserved in human H1 and HCT116 cells, although H3K27me3 enrichment in HCT116 cells is not limited to LAD borders (Additional file 1: Fig. S1E-F). Together, these data show that CTCF enrichment at LAD borders is conserved between cell types and that CTCF binding correlates with a different epigenetic makeup, and raise the question whether CTCF is involved in genome positioning at the NL.

### The majority of NL interactions are maintained upon depletion of CTCF

To test whether CTCF is directly involved in the demarcation of LAD borders, we used a mESC line with CTCF fused to an auxin-inducible degron (AID) tag [32]. Addition of auxin (IAA) results in rapid and near-complete protein depletion within several hours, allowing us to distinguish direct effects of CTCF depletion from secondary effects such as perturbed growth and cell differentiation [32]. We verified that IAA addition resulted in CTCF depletion (Additional file 1: Fig. S2A). Because the AID tag already resulted in a substantial decrease of CTCF levels even in the absence of IAA (Additional file 1: Fig. S2A), we used the parental clone (PT) expressing only OsTir1 as an additional control in these experiments.

We then used our recently developed pA-DamID procedure to map NL interactions at multiple time points after addition of IAA. Compared to conventional DamID, pA-DamID can map NL interactions with an increased temporal resolution [33]. The pA-DamID tracks generated in the parental line are very similar to DamID tracks in wildtype mESCs except for a reduced dynamic range (Additional file 1: Fig. S2B-C) and are highly reproducible between biological replicates (Additional file 1: Fig. S2D-E) and LAD borders called from pA-DamID tracks are equally enriched for CTCF binding (Additional file 1: Fig. S2F-G). Thus, the tracks generated with pA-DamID are in accordance with the DamID tracks and can be used to generate “snapshots” of NL interactions after protein depletion. To account for differences in dynamic range present between individual replicates and conditions, we converted log_2_ ratios of LaminB1/Dam-only to *z*-scores (Additional file 1: Fig. S2H). On average, a differential *z*-score of 1 corresponds to a 2.1-fold difference in the linear pA-DamID score (Additional file 1: Fig. S2I). The *z*-scores were averaged between biological replicates for downstream analyses [33].

NL interaction patterns are largely maintained after depletion of CTCF, but there are some small differences that gradually increase over time (Fig. 1D). As prolonged CTCF depletion can induce secondary effects in gene regulation and genome organization, we focused the following analyses on the early time points (6 h and 24 h). We observed no change in the cell cycle distribution after 6 h of CTCF depletion, but after 24 h, there is a weak depletion of S phase cells (Additional file 1: Fig. S3). The average profile of NL interactions across all combined LAD borders does not reveal large differences between CTCF-marked and CTCF-unmarked LAD borders in NL interactions (Fig. 1E). However, compared to unmarked borders, after CTCF depletion, we do observe a small but noticeable gain in NL interactions in the iLAD flanking CTCF-marked LAD borders (Fig. 1E, red arrows), precisely coinciding with the location of the CTCF binding sites (Fig. 1A). This change is already present after 6 h, suggesting that this is a direct effect of CTCF depletion. Quantification at individual LAD borders suggests a global increase at most borders rather than a strong effect at a small subset of borders (Fig. 1F). Approximately 25% of the CTCF-marked LAD borders shift ~20 kb into the iLAD after CTCF depletion (Fig. 1G).

Next, we performed similar analyses with different LAD border segmentations. First, we obtained comparable results for both CTCF orientations, suggesting that the observations are not caused by a directional loop extrusion process originating in either the LAD or iLAD (Additional file 1: Fig. S4A-C). Second, at LAD borders near active genes, CTCF depletion still induces a local gain at the CTCF binding site but does not affect the positioning of CTCF-marked borders (Additional file 1: Fig. S4D-F). It is thus likely that the active genes maintain the positioning of these borders. Third, to test if LAD borders with multiple CTCF binding sites are more dependent on CTCF, we segmented borders based on the amount of CTCF peaks within 20 kb. Indeed, we find that the number of CTCF binding sites positively correlates with the local gain outside of the LAD border (Fig. 1H) and the shift in LAD border positioning (Additional file 1: Fig. S4G-I). These data indicate that CTCF contributes to the sharpening of LAD borders, by counteracting DNA–NL contacts just outside of the borders, and thereby assists in LAD border positioning. However, we observe no dramatic change in global LAD patterns, indicating that CTCF is primarily involved in the fine-tuning of NL interactions.

### NL detachment at CTCF LAD borders is mediated by CTCF and cohesin

**Fig. 2:**
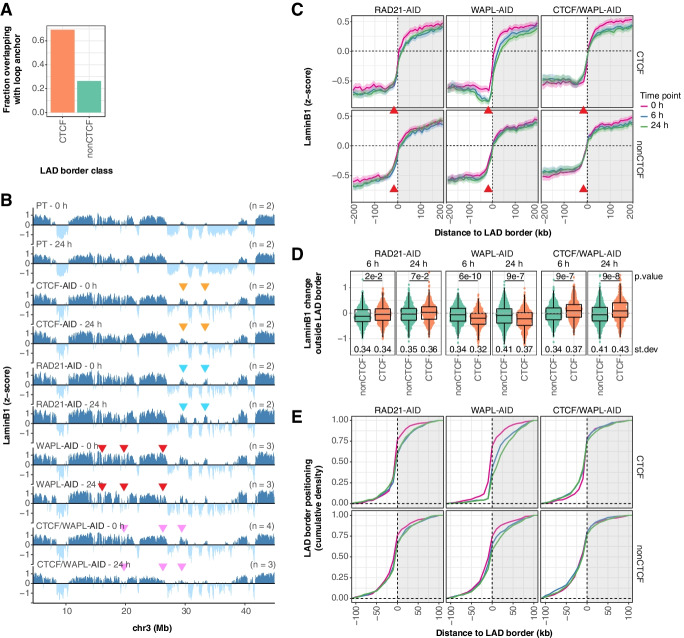
CTCF and cohesin mediate local detachment at CTCF-marked LAD borders. **A** Bar plot showing the fraction of LAD borders within 20 kb from a loop anchor as defined by ref. [36] **B** LaminB1 *z*-scores along a representative chromosome for all AID-tagged mESC lines without protein depletion (0 h) and following 24 h of IAA-induced protein depletion. Data are binned at 10 kb and averages of *n* biological replicates. The colored arrows highlight example regions with deviating LaminB1 signal over time and are described in more detail in the main text. **C** Average LaminB1 *z*-scores around LAD borders for protein depletion time courses (0 h, 6 h, 24 h) for mESCs with AID-tagged RAD21, WAPL, and a combination of CTCF and WAPL, as described in Fig. 1E. **D** Quantification of the change in LaminB1 *z*-score outside LAD borders, as described in Fig. 1F. **E** Cumulative density profile of LAD border positioning relative to LAD borders defined in the PT clone, as described in Fig. 1G

The looping function of CTCF is tightly linked to cohesin, a ring-shaped protein complex involved in the formation of long-range DNA contacts. Cohesin is stalled at CTCF binding sites in the genome and in turn is released from DNA by WAPL (reviewed in [48]). We observed that LAD borders with CTCF binding are enriched for RAD21 (Fig. 1C). Moreover, we find that CTCF-marked borders coincide more frequently with loop anchors than borders without CTCF [36] (Fig. 2A). These data together with the observation that WAPL knockout cells have fragmented LADs [25] suggest that cohesin may also be involved in LAD border organization. To directly test this, we mapped NL interactions in mESCs after rapid cohesin depletion (AID-tagged RAD21) [26], cohesin stabilization (AID-tagged WAPL) [26], and a combination of CTCF depletion and cohesin stabilization (AID-tagged CTCF and WAPL) [49] (Additional file 1: Fig. S2A). Similar to CTCF, AID-tagging of WAPL already leads to a substantial protein loss prior to addition of IAA, but WAPL is completely lost 6 h after IAA addition (Additional file 1: Fig. S2A). In accordance with a previous report, RAD21 depletion induces a strong enrichment of G2 cells [50] (Additional file 1: Fig. S3). The cell cycle distribution is virtually unaffected by WAPL depletion [26] or by a combined CTCF/WAPL depletion (Additional file 1: Fig. S3).

Similar to CTCF, these depletion experiments do not grossly alter the global patterns of NL interactions, although again various changes can be seen (Fig. 2B). Intriguingly, the changes differ strongly depending on the protein depleted. For example, RAD21 depletion results in changes that are sometimes opposite of those after CTCF depletion (orange and blue arrows). WAPL depletion rapidly fragments some LADs (red arrows), similar to a prolonged loss of WAPL [25]. A double depletion of WAPL and CTCF prevents LAD fragmentation, but does result in perturbed NL interactions for some LADs (pink arrows). All these changes that can be seen by visual inspection are likely caused by a combination of various mechanisms that we will discuss individually below.

Average profiles of NL interactions at LAD borders show that RAD21 depletion results in a small decrease in NL interactions around LAD borders marked by CTCF, both outside and inside the LAD (Fig. 2C, left panels). This likely reflects a global change in NL interactions rather than a local effect of cohesin at the LAD border. We do not observe a specific shift of CTCF-marked LAD borders compared to unmarked borders (Fig. 2E). A slight loss of the local dip in the average NL interaction profile at the position of CTCF binding sites (Fig. 2C, red arrows, Fig. 2D) seems to occur after RAD21 depletion, but this is largely masked by the overall decrease in NL interactions at CTCF-marked LAD borders.

More pronounced effects are seen after WAPL depletion (Fig. 2C, middle panels). The local dip in NL interactions just outside of CTCF-marked borders becomes much more pronounced (Fig. 2D), while these borders shift on average about 10–50 kb inwards (Fig. 2E). Remarkably, double depletion of WAPL and CTCF almost completely reverts this effect, underscoring that CTCF is required for cohesin-mediated local detachment (Fig. 2D, right panels). Under this double depletion condition, approximately 20% of the LAD borders with CTCF binding sites tend to shift slightly (~10 kb) into the iLAD (Fig. 2E).

Similar results are obtained for LAD borders bound by CTCF in either orientation (Additional file 1: Fig. S5A-B). We note that all observed changes in pA-DamID signals discussed above are caused by a change in LaminB1 reads rather than Dam control reads, illustrating that the observed differences are not due to changes in DNA accessibility (Additional file 1: Fig. S5C-D). We conclude that CTCF and cohesin can promote local detachment of DNA from the NL near LAD borders, which may help to reinforce these borders.

### Cohesin-mediated local detachment of CTCF-bound chromatin from the NL inside LADs

The data presented so far indicate that chromatin bound by CTCF can locally detach DNA from the NL at LAD borders. This raises the question whether this is specific for LAD borders, or also holds true for CTCF binding sites within LADs. Small euchromatin islands in large heterochromatin domains (typically overlapping with LADs [5]) are often devoid of a TSS and enriched for active marks (DNA accessibility and H3K9ac) and binding of CTCF [51]. However, it is unclear whether CTCF is required to locally escape from the NL at these sites.

**Fig. 3:**
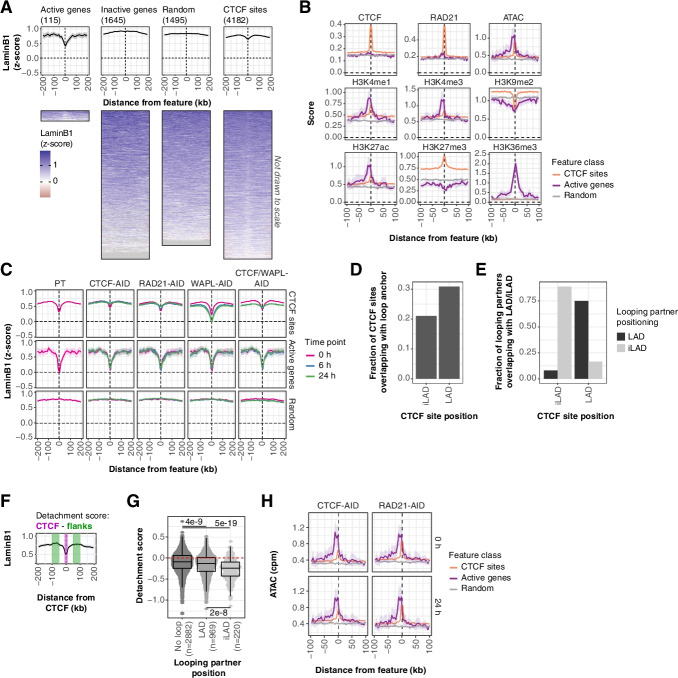
CTCF binding sites locally detach from the NL by cohesin-mediated long-range DNA interactions. **A** LaminB1 *z*-scores around active and inactive genes, CTCF binding sites, and random locations in LADs defined in the mESC PT clone, visualized as an average signal with 95% confidence interval of the mean (top panel) and heatmap (bottom panel). In the heatmap, rows represent individual sites and missing data is shown as gray tiles. Genes are filtered to be positioned at least 100 kb from CTCF binding sites, and vice-versa for CTCF binding sites. **B** Average scores around CTCF binding sites, active genes, and random locations in LADs for the epigenetic data sets, as described in Fig. 1C. **C** Average LaminB1 *z*-scores as in **A** following a protein depletion time course (0 h, 6 h, and 24 h) of CTCF, RAD21, WAPL, and a combination of CTCF and WAPL. Note that the PT data in panels **A** and **C** are from distinct sets of experiments; all data in **C** were obtained with the same batch of Lamin B1 antibody, which is different from the batch used for the data in **A**. The PT data from panel **C** lack an auxin-treated time point and are therefore not used in the other figures, but are better suited for direct comparisons with the untreated conditions (0 h) in the other clones in this panel (see also Additional file 1: Fig. S6A). **D** Similar plot to Fig. 2A, but showing the fraction of CTCF binding sites in iLADs and LADs that overlap with loop anchors. Loop anchor data is from ref. [36] **E** CTCF binding sites that overlap with loop anchors are classified based on LAD overlap. The bar plot shows the positioning of their looping partners in iLAD or LAD regions. **F** Illustration of the detachment score, defined as the difference between the LaminB1 score at the CTCF binding site and its 100-kb flanking region (see the “Methods” section). **G** The distribution of the detachment score (**F**) for CTCF binding sites, separated by loop anchor overlap and anchor positioning. CTCF binding sites within 100kb from LAD borders are excluded. A Wilcoxon test followed by Benjamini–Hochberg multiple testing correction was used to test for statistical significance. **H** Average counts-per-million (cpm) normalized ATAC-seq signals as in B for CTCF-AID and RAD21-AID cell lines without (0 h) and with (24 h) IAA-induced protein depletion

Consistent with previous work [11, 14, 52, 53], actively transcribed genes inside LADs locally detach from the NL in mESCs (Fig. 3A). Again, to avoid confounding effects from this expression-mediated detachment, we focused on CTCF binding sites that are positioned at least 100 kb away from active genes. We find that CTCF sites inside LADs moderately detach from the NL compared to the surrounding regions (Fig. 3A). Note that these detachments are not strong enough to cause our LAD-calling algorithm to mark these sites as iLADs.

Similar to LAD borders, CTCF binding in LADs is correlated with features of open chromatin (ATAC, H3K4me1, and H3K27ac), although to a much lesser extent than active genes. CTCF occupancy also coincides with a local decrease and increase of H3K9me2 and H3K27me3, respectively (Fig. 3B). These data are in accordance with our observations at LAD borders.

Average NL interaction profiles across active genes, CTCF sites, and random LAD locations show that CTCF and cohesin are required for local detachment of CTCF sites within LADs (Fig. 3C). This effect appears to be dose-dependent, as this detachment is slightly more pronounced in the PT line (with wild-type protein levels) compared to the clone with AID-tagged CTCF in the absence of IAA (which has a partial protein depletion) (Additional file 1: Fig. S6A). The changes in NL contacts are not observed upon treatment of PT cells with IAA and hence are not the result of an off-target effect of IAA (Additional file 1: Fig. S6B). Moreover, there is no change in NL interaction of active genes after any protein depletion (Fig. 3C), underscoring that the effects are specific for CTCF binding sites. WAPL depletion increases this local detachment of CTCF sites in a CTCF-dependent (Fig. 3C) and dose-dependent manner (Additional file 1: Fig. S6A). Together, these data indicate that CTCF- and cohesin-mediated detachment from the NL is not limited to LAD borders and that this involves a mechanism that is distinct from transcription-mediated detachment.

Next, we tried to better understand the local detachment of CTCF binding sites from the NL. We hypothesized that CTCF and cohesin together mediate long-range DNA interactions of CTCF binding sites with non-LAD regions, which then compete with NL contacts. Both within and outside LADs, CTCF binding sites frequently overlap with loop anchors (Fig. 3D). Loops are often formed within iLADs and within LADs, with limited cross-talk (Fig. 3E). We calculated a detachment score for every CTCF binding site in LADs, defined as the difference in NL interactions between the CTCF binding site and its flanking 100-kb regions (Fig. 3F). In accordance with our hypothesis, local detachment is correlated with the presence of a loop anchor (Fig. 3G), in particular when the looping partner is positioned outside of a LAD (Fig. 3G).

### Chromatin accessibility at CTCF binding sites is independent of NL detachment

Local NL detachment at CTCF binding sites with active chromatin features is thus dependent on CTCF and cohesin. This raises the question whether the detachment is required for the active chromatin features. To address this question, we generated ATAC-seq data to determine DNA accessibility [54] — one of the features enriched at CTCF binding sites — after depletion of CTCF. The results show that CTCF-mediated detachment is not required to maintain DNA accessibility (Fig. 3H). We obtained similar results after depletion of RAD21 (Fig. 3H). Combined, we conclude that CTCF-bound chromatin locally detaches from the NL, but that this is not required to maintain an accessible chromatin state.

### Architectural proteins control genome-wide patterns of NL interactions

**Fig. 4:**
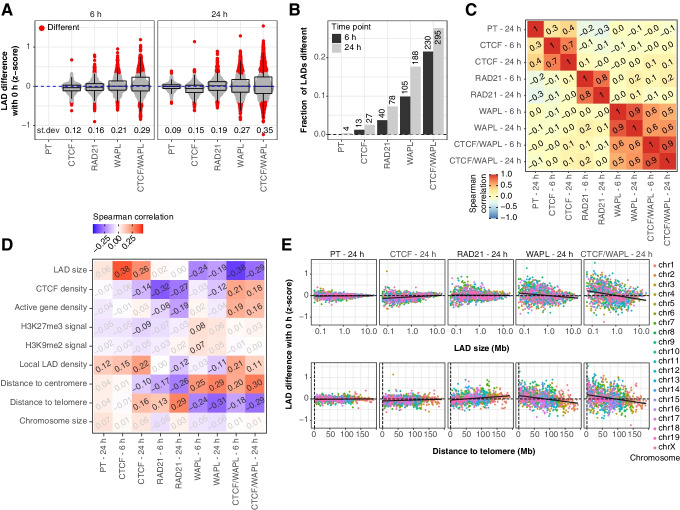
Cohesin dynamics affect chromosomal NL interaction patterns. **A** Quantification of the LAD differences in LaminB1 *z*-scores over time (Additional file 1: Fig. S7). The standard deviation is included. Changes that exceed the distribution observed for the PT 24 h control (beyond 0.1% and 99.9% of the data points) are classified as different. **B** Fraction of the LADs classified as different. The actual number of LADs is included. **C** Heatmap showing Spearman correlation coefficients between the LAD differences following auxin addition. LAD differences from the same depletion experiment are not independent as these compare differences relative to the same starting point, and are thus inflated. **D** Heatmap showing Spearman correlation coefficients between LAD features (Additional file 1: Fig. S8) and differences in LAD scores. Non-significant correlation (*p* > 0.05, using “cor.test()” in R) after Benjamini–Hochberg multiple testing correction is in gray. The LAD features are defined as follows: LAD size (log_2_ bp), CTCF binding and active gene density (count/Mb), average H3K27me3 and H3K9me2 signal, local LAD density (fraction of the surrounding 30 Mb classified as LAD), distance to chromosome ends (Mb), and size of the corresponding chromosome (Mb). **E** Scatterplots of LAD differences over time versus LAD size (top panels) and distance to telomere (bottom panels), colored by chromosome. The black line represents a fitted linear model

Up to this point, we analyzed local changes of NL interactions at CTCF binding sites. Next, we searched for more global effects of CTCF and cohesin on NL interactions, for example involving entire LADs or large chromosomal regions. Towards this goal, we calculated mean pA-DamID scores on a consensus set of LADs across all conditions and then determined the changes in scores in each depletion experiment (see the “Methods” section, Additional file 1: Fig. S7). We observed the smallest changes in LAD scores upon CTCF depletion and the biggest changes upon the double depletion of WAPL and CTCF (Fig. 4A). To quantify the fraction of affected LADs, we counted LADs that changed beyond the PT distribution. This revealed that up to 25% of the LADs are affected by the CTCF/WAPL depletion (Fig. 4B). Consistent with visual observations of the chromosome profiles illustrated previously (Fig. 2C), LAD-wide changes in NL interactions are mostly uncorrelated between the depleted proteins, except for WAPL and the double combination of CTCF and WAPL (Fig. 4C).

We then compared LAD-wide changes in NL interactions with several features that describe LADs and their chromosomal context: LAD size, density of CTCF binding and active genes, mean signal for the repressive histone modifications H3K27me3 and H3K9me2, local LAD density, distance to centromeres and telomeres, and chromosome size. Except for chromosomal positioning, most of these features are largely independent (Additional file 1: Fig. S8). This analysis revealed multiple significant correlations that strongly differ depending on the depleted proteins (Fig. 4D). For example, depletion of CTCF preferentially results in decreased NL interactions of small LADs, while loss of WAPL preferentially reduces NL interactions of large LADs (Fig. 4E, top panels). Furthermore, the effects of RAD21 and WAPL on LAD-wide NL interactions both depend on the chromosomal position (i.e., distance to telomere and centromere), but in opposite orientation. This effect appears roughly linear with distance to the telomeres and is present across all chromosomes (Fig. 4E, bottom panels). Chromatin nearby telomeres is enriched at the NL in early G1 cells and slowly loses interactions with the NL in interphase [33]. Possibly stabilized cohesin somehow prevents this normal maturation of NL interactions. It is unlikely that these observations are a direct consequence of a perturbed cell cycle, because the latter is only strongly affected by the RAD21 depletion (Additional file 1: Fig. S3).

We also found various correlations with local LAD density, CTCF density, and gene density (Fig. 4D). While these intriguing global links are currently difficult to interpret mechanistically, they illustrate that the CTCF/cohesin machinery affects NL interactions not only locally, but also at the scale of entire chromosomes. Interestingly, we found only few and very modest significant correlations with H3K9me2 and H3K27me3, suggesting that the effects of perturbed cohesin dynamics are largely independent of these histone modifications.

### Genome-wide changes in NL interactions are not mediated by transcription

Altered genome positioning at the NL is strongly correlated with transcriptional changes during differentiation [6]. This is particularly relevant here, as depletions of WAPL and RAD21 induce a differentiation-like phenotype [26]. To test whether the genome-wide effects on NL interactions are mediated by transcriptional changes, we analyzed RNA-seq data after all protein depletion experiments.

**Fig. 5:**
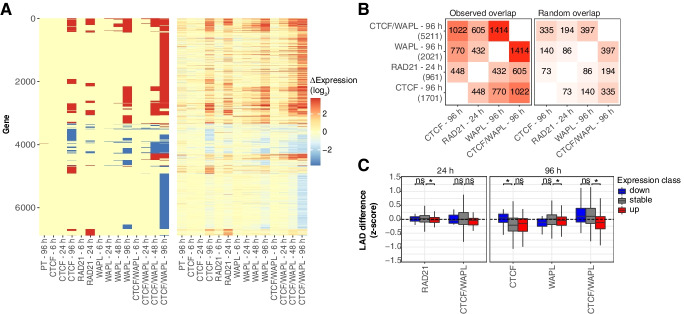
LAD dynamics are not caused by transcriptional effects of CTCF and cohesin perturbation. **A** Overview of RNA-seq results for time courses of CTCF depletion and cohesin perturbation, showing differentially expressed genes (left panel) and log_2_-fold changes (right panel) compared to 0 h. RNA-seq data in PT, RAD21, and WAPL clones are from ref. [26] **B** Comparison between the observed overlap in differentially expressed genes (left panel), versus a random overlap in differentially expressed genes (right panel). Only active genes (FPKM > 1) were considered for this comparison. **C** Distribution of LAD differences classified by the presence of up- and downregulated genes. LADs overlapping both up- and downregulated genes were excluded. Only conditions with at least 50 differentially expressed genes and available gene expression and pA-DamID data are shown. A Wilcoxon test was used to test for statistical significance, followed by Benjamini–Hochberg multiple testing correction (an asterisk denotes a *p*-value < 0.05)

Principal component analysis (PCA) (Additional file 1: Fig. S9A) and differential expression analysis (Fig. 5A, B) indicate that all four protein depletions have a partial overlap in their effect on gene expression, although the effects of RAD21 depletion and the double CTCF/WAPL depletion are more pronounced than those of individual depletion of CTCF or WAPL. We found that genes known to be differentially expressed genes during mESC differentiation into neural precursor cells [55] are significantly affected after all depletions (Additional file 1: Fig. S9B-C). This implies that both CTCF and dynamic cohesin are required to maintain a normal gene regulation program and prevent mESC differentiation [26]. However, we found that LADs with up- and downregulated genes do not consistently exhibit decreased and increased NL interactions, respectively (Fig. 5C). This is particularly true for LADs with early changes in gene expression (after 24 h of RAD21 and CTCF/WAPL depletions). We obtain similar results with nascent transcription data following WAPL depletion (Additional file 1: Fig. S9D) [26]. We conclude that the genome-wide changes in NL interactions are generally not mediated by transcriptional changes induced by the protein depletions.

We also asked whether LAD genes are specifically affected following cohesin perturbations. Most LAD genes are not expressed and their promoters are also not expressed when placed in a neutral chromatin environment [56]. Such genes may simply lack critical transcription factors and are thus unlikely to be upregulated by changes in chromatin architecture alone. To remove such unresponsive genes, we required LAD genes to have a minimum expression level in at least one of the conditions and selected a matching set of iLAD genes with a similar distribution of expression levels as these LAD genes (Additional file 1: Fig. S9E). For all depletion experiments, differentially expressed genes are not significantly enriched in LADs (Additional file 1: Fig. S9F). We conclude that CTCF and cohesin can affect gene regulation, but not preferentially in LADs.

### NL interactions are not dependent on H3K27me3

We find that H3K27me3 is locally enriched near CTCF binding sites positioned at LAD borders and within LADs. This is of interest, because in human fibroblasts CTCF and EZH2 (methyltransferase of H3K27me3) were both reported to be involved in the peripheral positioning of LAD fragments [12]. Furthermore, CTCF binding sites that overlap with H3K27me3 domains show a local increase in H3K27me3 signal after CTCF depletion in mESCs cultured in serum conditions [32]. Combined, these data may signify that a loss of CTCF is compensated by locally increased H3K27me3 and that a double depletion of CTCF and H3K27me3 is required to perturb LAD border positioning.

**Fig. 6:**
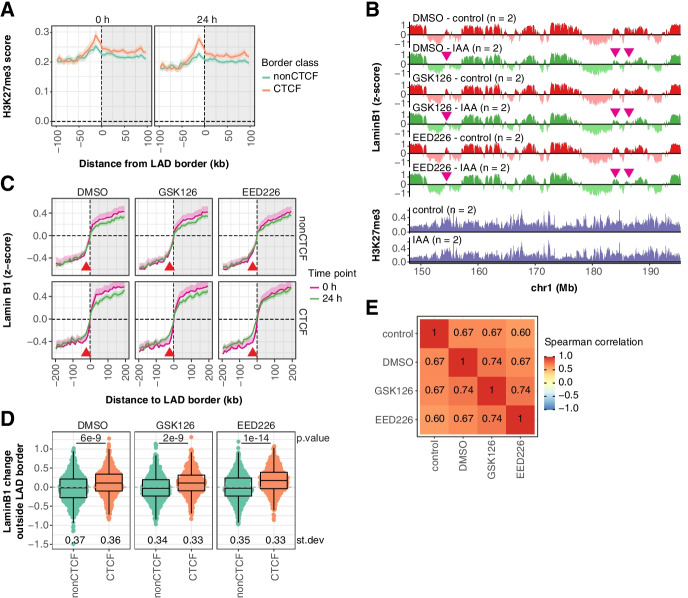
NL interactions are independent from H3K27me3 in mESCs. **A** Average H3K27me3 signal around mESC LAD borders, classified by CTCF presence. Human reads from spiked-in HEK293T cells were used to calibrate the mouse ChIP-seq data (Additional file 1: Fig. S10A, see the “Methods” section). Solid lines and shaded areas represent the mean signals and 95% confidence intervals of the mean, respectively. **B** Profile of LaminB1 *z*-scores along a representative genomic locus for CTCF-AID mESCs treated with DMSO and the H3K27me3 inhibitors GSK126 and EED226 (3 days) and with DMSO or IAA to induce CTCF depletion (last 24 h). Data are processed in 10-kb bins and are averages of *n* biological replicates. The pink arrows highlight example regions that lose LaminB1 signal upon CTCF depletion. Calibrated H3K27me3 ChIP-seq signals before and after CTCF depletion are added for comparison. **C** Average LaminB1 *z*-scores around LAD borders are shown for the samples in panel **B**, as described in Fig. 1E. **D** Quantification of the LaminB1 change outside LAD borders, as described in Fig. 1F. **E** Heatmap showing Spearman correlation coefficients between the IAA-induced LAD differences for untreated CTCF-AID cells (control) and for cells treated with DMSO and the H3K27me3 inhibitors

We first generated calibrated ChIP-seq data for H3K27me3 before and after CTCF depletion in mESCs cultured in 2i conditions. Alignment at LAD borders indicates that the enrichment of H3K27me3 is not affected by CTCF depletion; if anything, it becomes more pronounced (Fig. 6A). Next, to test the hypothesis that H3K27me3 and CTCF redundantly position LAD borders, we used two H3K27me3 methyltransferase inhibitors (GSK126 and EED226) that induce a near-complete loss of this mark (Additional file 1: Fig. S10B-C), and profiled NL interactions with and without CTCF depletion (Fig. 6B). H3K27me3 inhibition by itself does not affect global genome positioning at the NL (Additional file 1: Fig. S10D) or specifically at LAD borders with CTCF binding (Fig. 6C). Furthermore, a double depletion of H3K27me3 and CTCF has no additional effects besides those previously observed upon CTCF depletion alone (Fig. 6C–E).

We therefore conclude that there is no interplay between CTCF and H3K27me3 in genome positioning at the NL in mESCs. This result also suggests that the genome-wide correlation between NL interactions and H3K27me3 observed after CTCF depletion is not caused by the H3K27me3 marks (Fig. 4D), further supporting that the effects of perturbed cohesin dynamics on NL interactions are independent from histone modifications.

## Discussion

Combinations of chromosome conformation capture methods and rapid protein depletion experiments revealed how CTCF and cohesin organize the genome in self-associating domains [24, 25, 32, 57, 58]. However, it has remained mostly unclear how these processes affect nuclear positioning of the genome relative to the nuclear lamina. Here, we show that CTCF and cohesin reinforce LAD border positioning by mediating local detachment from the NL and affect genome positioning at the NL quantitatively. The observed effects of CTCF and cohesin on NL interactions appear to be relatively modest. Because the chromatin makeup of mESCs is quite unique compared to differentiated cells, the effects may be more or less pronounced in other cell types. It will be interesting to explore this in the future, using the combination of pA-DamID and degron-tagged proteins as described here.

### CTCF mediates local detachment from the NL

We find CTCF and cohesin enrichment at LAD borders in all tested mouse and human cell lines, indicating that this is a conserved feature of LADs [1, 12]. Our data in mESCs indicate that these proteins are not required to maintain LAD border positioning, but rather contribute to a sharper transition in NL interactions. This result complements previous work indicating that CTCF loss does not trigger spreading of H3K27me3 heterochromatin [32]. CTCF binding may still be a boundary for other types of heterochromatin, as H3K9me2 spreading was observed following CTCF depletion for a number of genes [59], but further studies are required to validate this. Furthermore, it remains to be elucidated whether there is a functional impact of a sharper transition in NL interactions.

Small euchromatin regions inside heterochromatin blocks can be divided in promoter elements and regulatory elements based on genomic features and chromatin marks, both of which are enriched for CTCF binding sites [51]. This is in accordance with our results, which indicate that isolated CTCF sites in LADs are enriched for active marks and locally detach from the NL. We show that NL detachment at these sites requires both CTCF and cohesin. Thus, besides transcription [11, 14, 52, 53], DNA looping mediated by CTCF and cohesin can contribute to the local dissociation of DNA from the NL. Upon cohesin stabilization, this CTCF-mediated detachment can fully fracture LAD domains and thus establish new LAD borders [25]. Our data reveal a stronger detachment for CTCF sites that loop with iLAD regions. This result suggests that extrusion can pass LAD borders and upon loop stabilization mediate focal dissociation of specific LAD sequences towards the nuclear interior.

Intriguingly, CTCF and cohesin are dispensable for DNA accessibility at CTCF binding sites in LADs. This is of interest, as artificial decondensation of LADs can induce repositioning from the NL, even without transcriptional activity [7, 11, 60]. While DNA accessibility does not directly mirror chromatin decondensation, it may indicate that this repositioning also depends on CTCF and cohesin. Alternatively, these observations could be explained by a mechanism independent from transcription and CTCF-mediated DNA looping to detach from the NL.

### Cohesin dynamics affect genome-wide patterns of NL interactions

Besides local effects at LAD borders and CTCF binding sites, CTCF and cohesin affect the genome-wide pattern of NL positioning quantitatively. We observe the largest effects upon depletion of WAPL and combined depletion of CTCF and WAPL. While these genome-wide changes are difficult to interpret mechanistically, they reveal intriguing correlations. For example, cohesin stabilization disrupts positioning of telomeres and nearby DNA in the nuclear interior, which is a characteristic of genome reorganization in early interphase [33, 61]. While we do not find specific effects on gene expression in LADs, it remains unclear whether other heterochromatic phenotypes such as telomere maintenance and replication timing are affected by nuclear repositioning following perturbed cohesin dynamics [8, 62].

It was previously reported that CTCF is required for the peripheral positioning of integrated LAD fragments [12]. This generally contradicts with the small effects that we observe upon CTCF depletion. We speculate that this strong effect is a consequence of the small size of the LAD fragment, which in our data correlates with decreasing NL interactions. Mixing of nearby chromatin — typically inhibited by CTCF [32, 63] — may drag small, isolated LADs into the nuclear interior.

A similar dependency for the peripheral position of LAD fragments was shown for EZH2, the methyltransferase responsible for H3K27me3 deposition [12]. This differs from our results, which show that inhibition of EZH2 (with GSK126) does not affect NL interactions, alone or combined with CTCF depletion. Possibly, this discrepancy reflects cell type differences in NL affinity. H3K27me3 (and presumably also EZH2) is enriched inside LADs for some cell types, such as HCT116 cells, but not for mESCs (Fig. 1C, Additional file 1: S1F). In accordance with this reasoning, peripheral positioning of H3K27me3 is a transient phenotype during the cell cycle, development, and senescence [28, 29, 64].

Previous work illustrated that chromatin positioning at the NL is not required for genome compartmentalization [65]. Vice-versa, our data suggests that genome compartmentalization, which is strongly and rapidly weakened upon WAPL and CTCF/WAPL depletion [26, 49], is also not required for NL positioning.

### What then determines LAD border positioning?

Overall, our data indicate that CTCF and cohesin are not required for LAD border positioning in mESCs, but only contribute to sharpening of the borders. It thus remains to be elucidated what demarcates LADs. While we cannot rule out that an undiscovered factor is involved, it is also possible that there are no specific characteristics of LAD borders. Rather, the factors that mediate NL interactions may be tightly controlled and result in sharp LAD borders.

Constitutive LADs and their borders are conserved between human and mouse cells [66]. While the nucleotide sequences are highly divergent, both species maintain a high AT-content in these LAD domains that presumably mediates interactions with specific NL components [66]. This may involve H3K9me2, which is evolutionary conserved, is retained throughout mitosis, and quickly re-establishes peripheral positioning in daughter cells [9]. It is, however, likely that additional mechanisms play a role as well. For example, similar to constitutive LADs, mESC-specific LADs have a high AT-content [66]. This is in accordance with a model that LADs in stem cells reflect a “basal” state of chromosome organization, and suggests that AT-rich DNA somehow can be targeted to the NL. During differentiation, the basal organization could then be rearranged by a different NL composition that interacts with chromatin marks such as H3K27me3. In this model, CTCF, cohesin, and transcription are not integral to the general pattern of LAD domains. Instead, these factors can modulate NL interactions via forces, such as the formation of loops, that counteract peripheral positioning.

## Conclusion

We systematically investigated how NL interactions are affected by proteins involved in the loop extrusion process. Remarkably, while cohesin perturbation drastically affects genome compartmentalization, NL interaction patterns are largely unaffected. Rather, CTCF and cohesin promote focal detachment from the NL and affect peripheral positioning of the genome quantitatively.

## Methods

### Experimental procedures

#### Cell culture

E14Tg2a mouse embryonic stem cells (mESCs) (129/Ola isogenic background) and derived clones were cultured in serum-free DMEM/F12 (Gibco) and Neurobasal (Gibco) medium (1:1), supplemented with N-2 (Gibco), B-27 (Gibco), BSA (0.05%; Gibco), 104 U of leukemia inhibitory factor (LIF; Millipore), MEK inhibitor PD0325901 (1 μM; Selleckchem), GSK-3β inhibitor CHIR99021 (3 μM; Cayman Chemical), and 1-thioglycerol (1.5 × 10−4 M; Sigma-Aldrich) on 0.1% gelatin-coated plates. Cells were passaged every 2 days. Cells were seeded and incubated overnight before starting protein depletion experiments, at the following densities: for a 96-h time course, 35,000 and 150,000 cells were seeded in 6-well and 10-cm plates, respectively; for a 24-h time course, 0.5 million and 1.5 million cells were seeded in 6-well and 10-cm plates, respectively. During these time courses, the medium was refreshed or cells were split 1:10 every 2 days.

Protein depletion was induced by treating cells with a final concentration of 500 μM auxin (IAA) (I5148-10G, Sigma-Aldrich). Time series experiments were performed by inducing protein degradation at different time points and collecting all samples at the end of the time course.

F121-9-CASTx129 mESC were cultured in 2i conditions according to the 4D Nucleome guidelines (https://data.4dnucleome.org/biosources/4DNSRMG5APUM/). These cells were differentiated into neural precursor cells (mNPCs) and cultured as described [6].

Cells were tested for mycoplasma every 3 months.

#### Western blots

To verify protein depletion, AID-tagged mESCs were treated for 6 h with H_2_O or IAA and harvested. To obtain the nuclear soluble fraction, cells were resuspended in 500 μL of low salt buffer (final concentration: 10 mM HEPES, 50 mM NaCl, 1 mM EDTA, 1mM DTT, and 1X Protease Inhibitor Cocktail (11697498001, Sigma-Aldrich)) and incubated on ice for 15 min, followed by adding 500 μL of low salt/0.4% NP-40 buffer to reach a final concentration of NP-40 at 0.2%. These cells were then rotated at 4 °C for 15 min and centrifuged at 6000 g for 10 min at 4 °C. The obtained cell pellets were resuspended in high salt buffer (final concentration: 10 mM HEPES, 420 mM NaCl, 1 mM EDTA, 10% glycerol, and 1X Protease Inhibitor Cocktail) at a density of 5 million cells per 100 μl buffer and then rotated overnight at 4 °C. The supernatant was collected for subsequent experiments. For the whole cell lysates, the cells were lysed in RIPA lysis buffer (150 mM NaCl, 1% NP-40, 0.5% sodium deoxycholate, 0.1% SDS, and 25 mM Tris (pH 7.4)).

The nuclear extracts were used for western blot analysis of CTCF, WAPL, and ACTB, and the whole cell lysates were used for western blot analysis of RAD21 and HSP90. Precast gradient SDS-PAGE gels (NuPAGE^TM^ 4 to 12%, Bis-Tris 1.0 mm, Mini Protein Gel, 15-well, NP0323BOX, ThermoFisher SCIENTIFIC) were used to separate the proteins and transferred to preactivated PVDF membranes on a Trans-Blot Turbo Transfer System (Bio-Rad). The blots were incubated with primary antibodies against the following proteins overnight at 4 °C: (1) CTCF (1:1000; 07-729, Merck Millipore), (2) WAPL (1:1000; 16370-1-AP, Proteintech), (3) ACTB (1:5000; ab8227, Abcam), (4) RAD21 (1:1000; ab154769, Abcam), and (5) HSP90 (1:2000; 13171-1-AP). After incubation, the blots were washed three times with 0.1% Tween-20 in TBS (TBST) and then incubated with secondary antibody against rabbit IgG at room temperature for 1 h, again followed by three washes with TBST.

For H3K27me3 detection, mESCs were collected and lysed in lysis buffer (Tris pH 8.0 50 mM, EDTA 1mM, SDS 1 %) and sonicated. A total of 25 μg of total protein extracts was run on a gradient (15-4%) Polyacrylamide gel (MINIi-PROTEAN TGX Precast Gels, Biorad). Proteins were transferred on the nitro-cellulose membrane and checked with Red Ponceau staining for efficient transfer. The membranes were incubated with blocking buffer (5% milk powder in TBST) for 1 h at room temperature and afterwards incubated with primary antibody (anti-H3K27me3, Diagenode # C15410195, 1:1000) in blocking buffer overnight at 4 °C. Membranes were washed with TBST and secondary antibody incubation was performed for 2 h at room temperature in blocking buffer.

Protein detection was performed using Biorad Clarity Max ECL substrates and a ChemiDoc MP Imaging System (Bio-Rad).

#### Cell cycle analysis

Cell cycle analysis was performed with the Click-iT EdU Alexa Fluor 647 Flow Cytometry Assay Kit (Invitrogen). Briefly, for 1.5 h, mESCs were incubated with 10 μM Click-iT EdU and fixed and permeabilized. Cells were incubated with the Click-iT Plus reaction cocktail for 30 min at room temperature (protected from light) to detect EdU and stained with 4′,6-diamidino-2-phenylindole (DAPI). DAPI and EdU signals were quantified on a BD LSRFortessa analyzer and processed with FlowJo 10.3.

#### LaminB1 DamID and pA-DamID

LaminB1 DamID was performed as described previously [56, 67]. Briefly, F121-9-CASTx129 mESCs and mNPCs were lentivirally transduced in a 6-well plate with Dam control or (mouse) Dam-LaminB1 constructs. Dam was under the control of the human PGK1 promoter and fused to a destabilization domain. Genomic DNA was isolated 3 days after transduction (ISOLATE II Genomic DNA kit, Bioline BIO-52067), without stabilization of the Dam fusions to keep expression at low levels. ^m6^A-marked DNA was enriched using a sequence of DpnI digestion, PCR adapter ligation, DpnII digestion, and PCR amplification. The resulting amplified material was processed for high-throughput sequencing using an in-house library preparation procedure and sequenced for single-end 140-bp reads on an Illumina HiSeq 2500. Approximately 10 million reads were sequenced for every condition.

pA-DamID LaminB1 maps were generated as described [33]. One million E14Tg2a mESCs were collected by centrifugation (500 g, 3 min) and washed sequentially in ice-cold PBS and digitonin wash buffer (DigWash) (20 mM HEPES-KOH pH 7.5, 150 mM NaCl, 0.5 mM spermidine, 0.02% digitonin, cOmplete Protease Inhibitor Cocktail). Cells were rotated for 2 h at 4 °C in 200 μL DigWash with 1:400 Lamin B1 antibody (Abcam, ab16048, rabbit), followed by a wash step with DigWash. This was repeated with a 1:200 pA-Dam solution (~60 NEB Dam units), followed by 2 wash steps. Dam activity was induced by an incubation for 30 min at 37 °C in 100 μL DigWash supplemented with 80 μM SAM while gently shaking (500 rpm). Genomic DNA was isolated and DNA was processed similar to DamID, except that the DpnII digestion was omitted and 65-bp reads were sequenced. For every condition, another 1 million cells were processed in only DigWash and during Dam activation incubated with 4 units of Dam enzyme (NEB, M0222L). This Dam control sample serves to account for DNA accessibility and amplification biases.

#### ATAC-seq

ATAC-seq libraries were prepared as previously described [68]. Cells were permeabilized and tagmented with in-house-generated Tn5 transposase, after which DNA fragments were amplified with two sequential nine-cycle PCR runs. Fragments smaller than 700 bp were selected with SPRI beads. ATAC-seq libraries were sequenced with paired-end mode using a 75-cycle kit on an Illumina NextSeq 550.

#### ChIP-seq

ChIP-seq was performed as described but with small modifications [68]. mESCs and mNPCs were mixed with 10% HEK293T cells (as internal reference) and crosslinked for 10 min with 1% FA. The reaction was quenched with 2.0 M glycine. After cell lysis, a Bioruptor Plus sonication device (Diagenode) was used to fragment chromatin to approximately 300-bp fragments. Antibodies against CTCF (07-729, Merck Millipore; 5 μL per ChIP) or H3K27me3 (PAB-195-050, Diagenode; 5 μL per ChIP) were coupled with Protein G beads (Thermo Fisher Scientific) and incubated with fragmented chromatin overnight at 4 °C. After washing, chromatin was eluted from the beads and crosslinking was reversed. Released DNA fragments were purified with the MinElute PCR Purification kit (Qiagen). The purified DNA fragments were prepared with the KAPA HTP Library Preparation kit (Roche) and single-end sequenced with a 65-cycle kit on an Illumina HiSeq 2500 (CTCF) or a 75-cycle kit on a NextSeq 550 (H3K27me3).

#### RNA-seq

RNA was isolated using a standard TRIzol RNA isolation protocol (Ambion). Cells were lysed with 1 mL of TRIzol reagent, after which 200 μL chloroform was added and the mixture was vortexed. Following centrifugation at 12,000 g at for 15 min at 4 °C, the upper phase was homogenized with 0.5 mL of 100% isopropanol. Samples were incubated at room temperature for 10 min and centrifuged at 4 °C for another 10 min. The resulting RNA pellet was washed with ice-cold 75% ethanol, dried at room temperature, and resuspended in RNase-free water. The isolated RNA was treated with DNase using the RNeasy Mini kit (Qiagen). RNA-seq libraries were prepared using a TruSeq Stranded RNA LT Kit (Illumina). The libraries were sequenced for single-end 65-bp reads on an Illumina HiSeq 2500.

### Computational analyses

#### DamID and pA-DamID

DamID and pA-DamID data was processed as described [56]. Briefly, the adapter sequence was trimmed with cutadapt 1.11 before mapping the remaining genomic DNA sequence to mm10 with bwa mem 0.7.17. The following steps were performed with custom R scripts. Reads with a mapping quality of at least 10 and overlapping the ends of a GATC fragment were counted in 10-kb genomic bins. Counts were normalized to 1 million reads per sample and a log_2_-ratio over the Dam control sample was calculated with a pseudo count of 1. At least 2 biological replicates were generated for every experimental condition and the average score was used for downstream analyses. Log_2_ ratios were converted to *z*-scores to correct for differences in dynamic range between experiments (Additional file 1: Fig. S2H-I).

#### LAD definition

LADs were determined using hidden Markov modeling on the average NL interaction profile between biological replicates (https://github.com/gui11aume/HMMt). For Fig. 1A–C, the LAD definition based on wildtype mESC (F121-9-CASTx129 strain) LaminB1 DamID was used. For the remaining analyses, the LAD definition was based on PT mESC (E14Tg2a strain) LaminB1 pA-DamID, except when otherwise stated. In order to capture newly formed LADs, a consensus LAD model between all experimental conditions was used for Figs. 4, 5, and 6 (a union set of LADs called in PT and AID-tagged cell lines up to 24 h of IAA addition).

#### LAD border classification

LAD borders were classified as CTCF borders if a CTCF binding site was within 20 kb outside the LAD (overlapping with the enriched CTCF density). Borders within 10 kb of an active gene (FPKM > 1) were flagged and not used in downstream analyses unless otherwise indicated. LAD borders were assigned to a loop anchor if this was positioned within 20 kb. To determine CTCF orientation at LAD borders, the CTCF motif (JASPAR MA0139.1 [69]) was used to infer the orientation of CTCF binding sites with FIMO (MEME suite) [70]. LAD borders with a single CTCF orientation were assigned this orientation, while LAD borders near multiple CTCF orientations or unassigned CTCF binding sites were assigned “ambiguous.” Deeptools 3.5.0 [71] and custom R scripts were used to calculate and visualize scores relative to LAD borders and LAD features (i.e. active genes, CTCF binding sites).

#### Nuclear lamina detachment score

The detachment score was defined as the difference in mean NL interaction scores between the flanking region (distances between 50 and 100 kb, both sides) and the CTCF binding site (up to 10 kb). To prevent confounding factors for transcription and border positioning, CTCF binding sites were filtered to be at least 100 kb from active genes and LAD borders.

#### RNA-seq and TT-seq

RNA-seq reads were mapped against the mm10 reference genome with TopHat2 2.1.1 [72] and filtered for a mapping quality of at least 10. Read counts for Ensembl genes (GRCm38.92; exons only) were determined with HTSeq 0.9.1. DESeq2 1.30.1 [73] was used to call differentially expressed genes by testing for a log_2_-fold difference of 0.5 with a false discovery rate of 0.05. Principal component analysis was performed with “plotPCA()” from DESeq2, using the top 5000 most variable genes. FPKM values were calculated with “fpkm()” from DESeq2, using the combined exon length as gene length. Similar downstream processing was applied to call differential expression in the TT-seq data [26].

#### ATAC-seq

ATAC-seq reads were mapped to mm10 with bwa mem 0.7.15-r1140. SAMtools 1.9 [74] was used to filter reads for a mapping quality of at least 15 and discard optimal PCR duplicates. Genomic coverage was determined with deeptools 3.0 [71].

#### ChIP-seq

ChIP-seq data were analyzed as previously reported [26]. Reads were mapped to a concatenated reference genome (mm10 and hg19) using bowtie2 2.3.4.1 [75]. Reads were filtered for a mapping quality of at least 15 and optical PCR duplicates were removed with SAMtools 1.9. The normalization factor to scale the spike-in HEK293T reads (mapping to hg19) to 1 million reads was used as the scaling factor in deeptools 3.0 to determine genomic coverage. Peak calling was performed with MACS2 2.1.1.20160309 [76] at a *q*-value cutoff of 0.01.

#### External data

The external datasets that have been used in this study have been referred to in the main text and figure legends. Data identifiers are listed in Additional file 2: Table S1.

## Supplementary Information

Additional file 1: Fig S1. LAD border enrichment of epigenetic marks is independent of CTCF orientation and is conserved between cell types. Fig S2. LaminB1 pA-DamID tracks recapitulate DamID data and can be used to profile NL interactions after rapid protein depletion. Fig S3. Effect of protein depletions on the cell cycle. Fig S4. Further analysis of the effect of CTCF depletion on LAD border positioning. Fig S5. Perturbed NL interactions are limited for outwards oriented LAD borders and caused by changes in LaminB1 reads. Fig S6. Protein depletion by AID-tagging induces a partial effect on CTCF detachment within LADs. Fig S7. Quantification of LAD differences after CTCF depletion and cohesin perturbation. Fig S8. Correlation between LAD features. Fig S9. CTCF depletion and cohesin perturbation affect an overlapping gene set enriched for differentiation genes. Fig S10. H3K27me3 depletion does not affect genome-wide NL interactions.

Additional file 2: Table S1. List of datasets used.

Additional file 3. Review history

## Data Availability

The genomic datasets used in this article [77–127] are publicly available, as specified in Additional file 2: Table S1. The computational code used to analyze the data are available at 10.5281/zenodo.6977460 [128]. Laboratory notes of most laboratory experiments are available at https://osf.io/jw74g/ [129].
