# Supplementary material for: CTCF and cohesin promote focal detachment of DNA from the nuclear lamina
Source: Genome Biol. 2022 Sep 1;23:185. doi: 10.1186/s13059-022-02754-3 (PMC9438259; doi:10.1186/s13059-022-02754-3)
Supplement: Supplementary file 1 — Additional file 1: Fig S1. LAD border enrichment of epigenetic marks is independent of CTCF orientation and is conserved between cell types. Fig S2. LaminB1 pA-DamID tracks recapitulate DamID data and can be used to profile NL interactions after rapid protein depletion. Fig S3. Effect of protein depletions on the cell cycle. Fig S4. Further analysis of the effect of CTCF depletion on LAD border positioning. Fig S5. Perturbed NL interactions are limited for outwards oriented LAD borders and caused by changes in LaminB1 reads. Fig S6. Protein depletion by AID-tagging induces a partial effect on CTCF detachment within LADs. Fig S7. Quantification of LAD differences after CTCF depletion and cohesin perturbation. Fig S8. Correlation between LAD features. Fig S9. CTCF depletion and cohesin perturbation affect an overlapping gene set enriched for differentiation genes. Fig S10. H3K27me3 depletion does not affect genome-wide NL interactions. [file 13059_2022_2754_MOESM1_ESM.pdf]

CTCF and cohesin promote focal detachment of DNA from the nuclear lamina

Tom van Schaik[1], Ning Qing Liu[1], Stefano G. Manzo[1], Daan Peric-Hupkes[1,2], Elzo de Wit[1] and Bas van Steensel[1,3,4]

[1]Oncode Institute and Division of Gene Regulation, Netherlands Cancer Institute, Amsterdam, the Netherlands
[2]Current address: Annogen, Amsterdam, the Netherlands
[3]Department of Cell Biology, Erasmus University Medical Center, Rotterdam, the Netherlands
[4]Corresponding author

Additional file 1: Supplementary figures S1-10

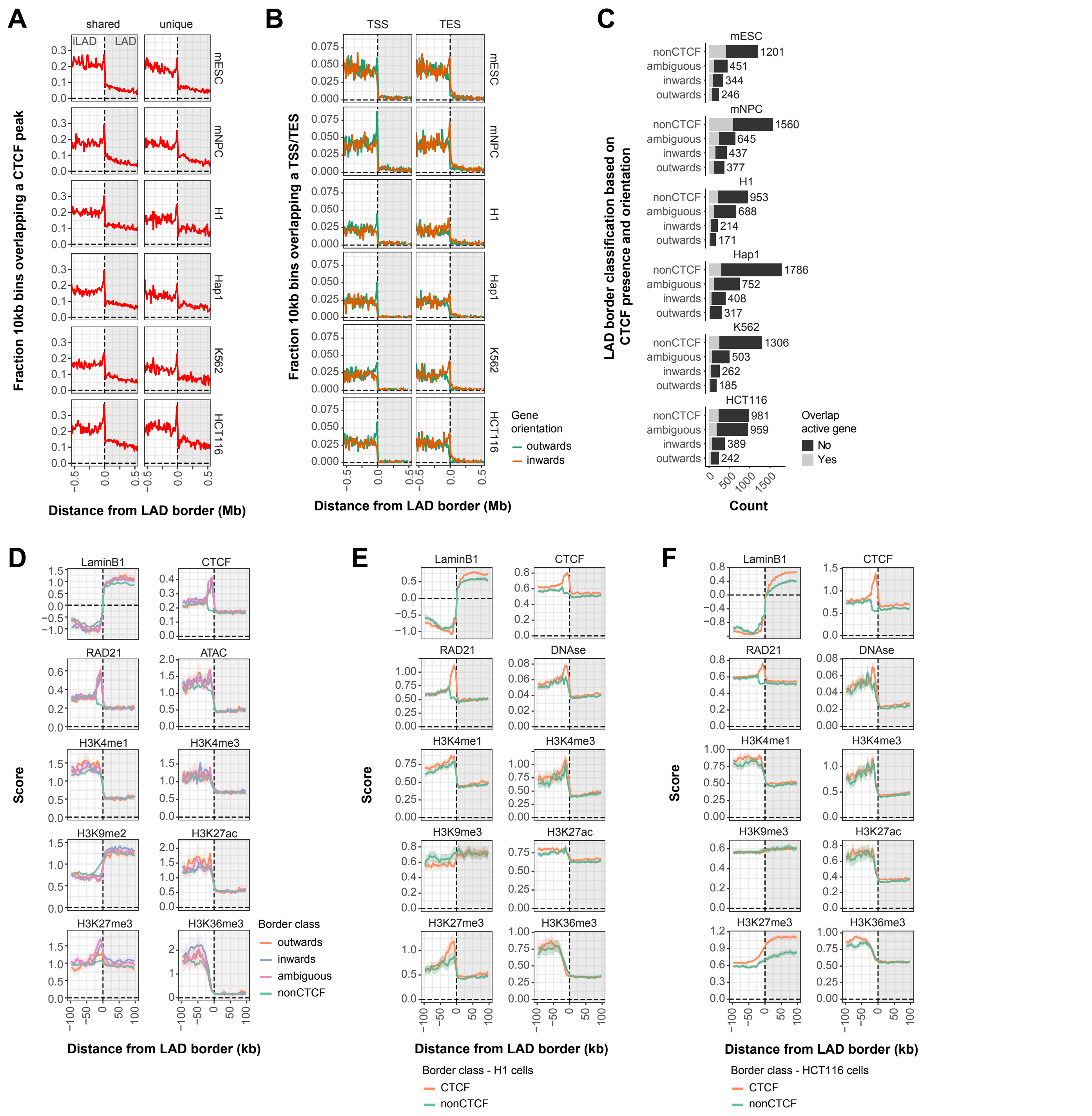

Fig S1. LAD border enrichment of epigenetic marks is independent of CTCF orientation and is conserved between cell types

(A) Profiles similar to (Fig. 1A), showing CTCF enrichment around LAD borders separated by conservation between cell types. LAD borders are shared between cell types when a LAD border in another cell type of the same species is within 100 kb. **(B)** Transcription start site (TSS) and transcription end site (TES) enrichment of active genes (FPKM > 1) is shown around LAD borders. Gene orientation was used to determine the orientation relative to the LAD, where inwards and outwards indicate a gene transcribing towards and away from the LAD, respectively. **(C)** Classification of LAD borders similar to (Fig. 1B), but including CTCF motif orientation in the LAD border classification. LAD borders overlapping with CTCF binding sites for which the orientation could not be determined and borders overlapping with multiple orientations were classified as ambiguous LAD borders. Significantly more CTCF binding sites have been called in H1 and HCT116 cell lines (55-60x10^3 compared to 30-45x10^3), resulting in a larger fraction of ambiguous LAD borders for these cell lines. **(D)** Profiles similar to (Fig. 1C), but using LAD borders classified with CTCF motif orientation. **(E-F)** Profiles similar to (Fig. 1C), but for LAD borders in H1 (E) and HCT116 (F) cells. Due to data availability and data quality, ATAC-seq was replaced by DNAse as a measure for DNA accessibility and H3K9me2 was replaced by H3K9me3 in both cell types. Data are from ref [35].

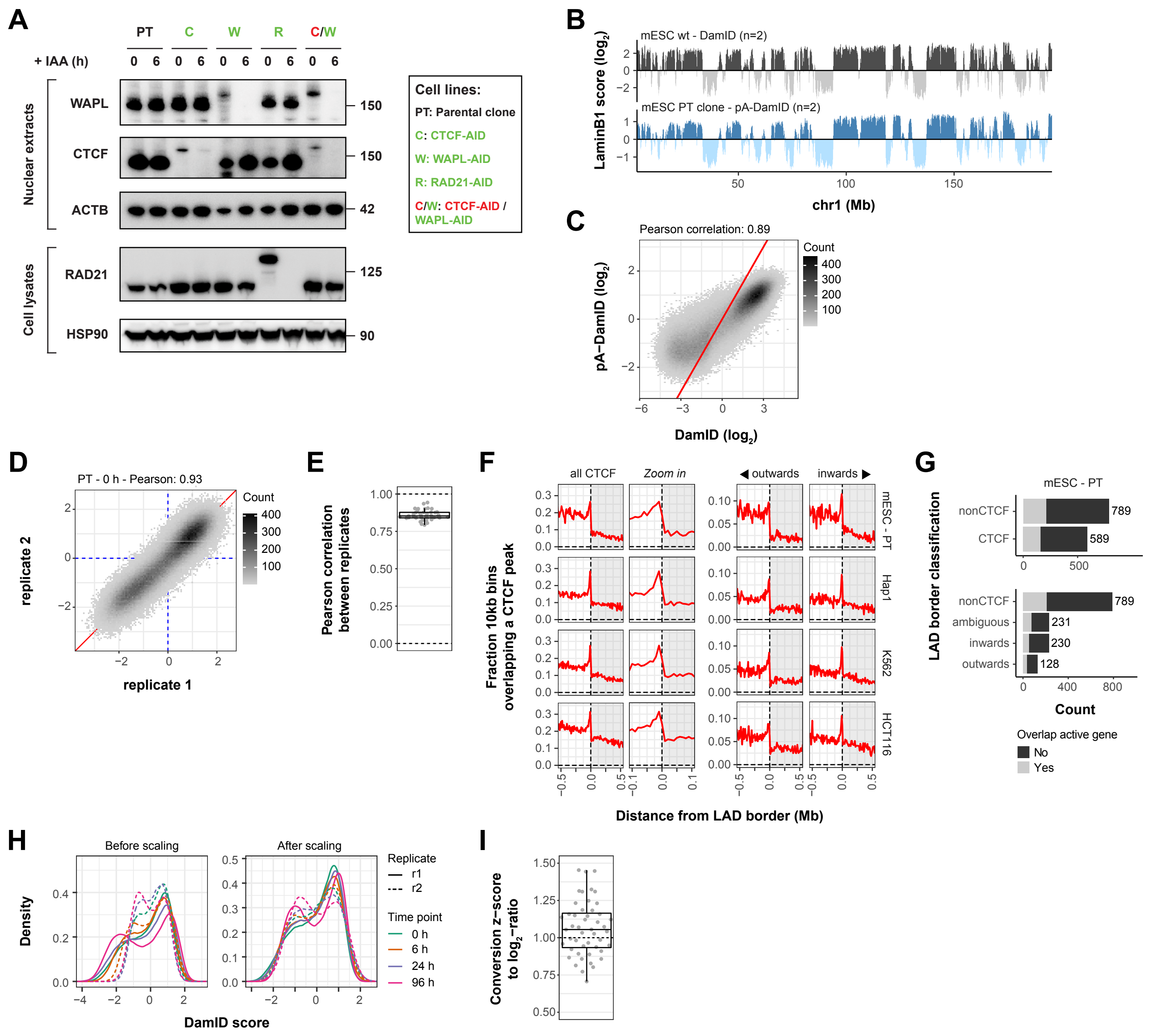

Fig S2. LaminB1 pA-DamID tracks recapitulate DamID data and can be used to profile NL interactions after rapid protein depletion

(A) Western blot analysis of WAPL, CTCF and RAD21 levels in the parental line (PT) and AID-tagged mESC clones before and after 6 hours of IAA treatment (cropped, n = 1). Green and red text indicate AID-fusions with GFP and mCherry, respectively. **(B)** Profile of LaminB1 interaction scores along a representative chromosome generated with DamID (in wildtype mESCs, F121-9 strain) and pA-DamID (in mESC PT clone, E14Tg2a strain). The $\log_2$-ratios of LaminB1 reads over Dam-control reads are shown for 10 kb genomic bins. Data tracks are averages of *n* biological replicates. **(C)** Scatterplot of LaminB1 $\log_2$-ratios from (B) for all chromosomes. The red line represents the diagonal. **(D)** Scatterplot of LaminB1 pA-DamID interaction scores ($\log_2$-ratios) for two PT clone replicates prior to IAA addition. The red line represents the diagonal. **(E)** Distribution of Pearson correlation values between all biological replicate experiments described in this manuscript. **(F)** Profiles similar to (Fig. 1A), showing CTCF enrichment around LAD borders based on pA-DamID data in the mESC PT clone. **(G)** LAD border classification plots similar to (Fig. 1B) and (Fig. S1C), for LADs defined with pA-DamID data in the mESC PT clone. **(H)** Data distribution of LaminB1 pA-DamID $\log_2$-ratios before (left panel) and after (right panel) conversion to z-scores for the CTCF-AID clone. The distribution shapes are not affected by this transformation. Every line represents a single replicate. **(I)** Distribution of the scaling factors to transform z-scores to $\log_2$-ratios for all experiments described in this manuscript.

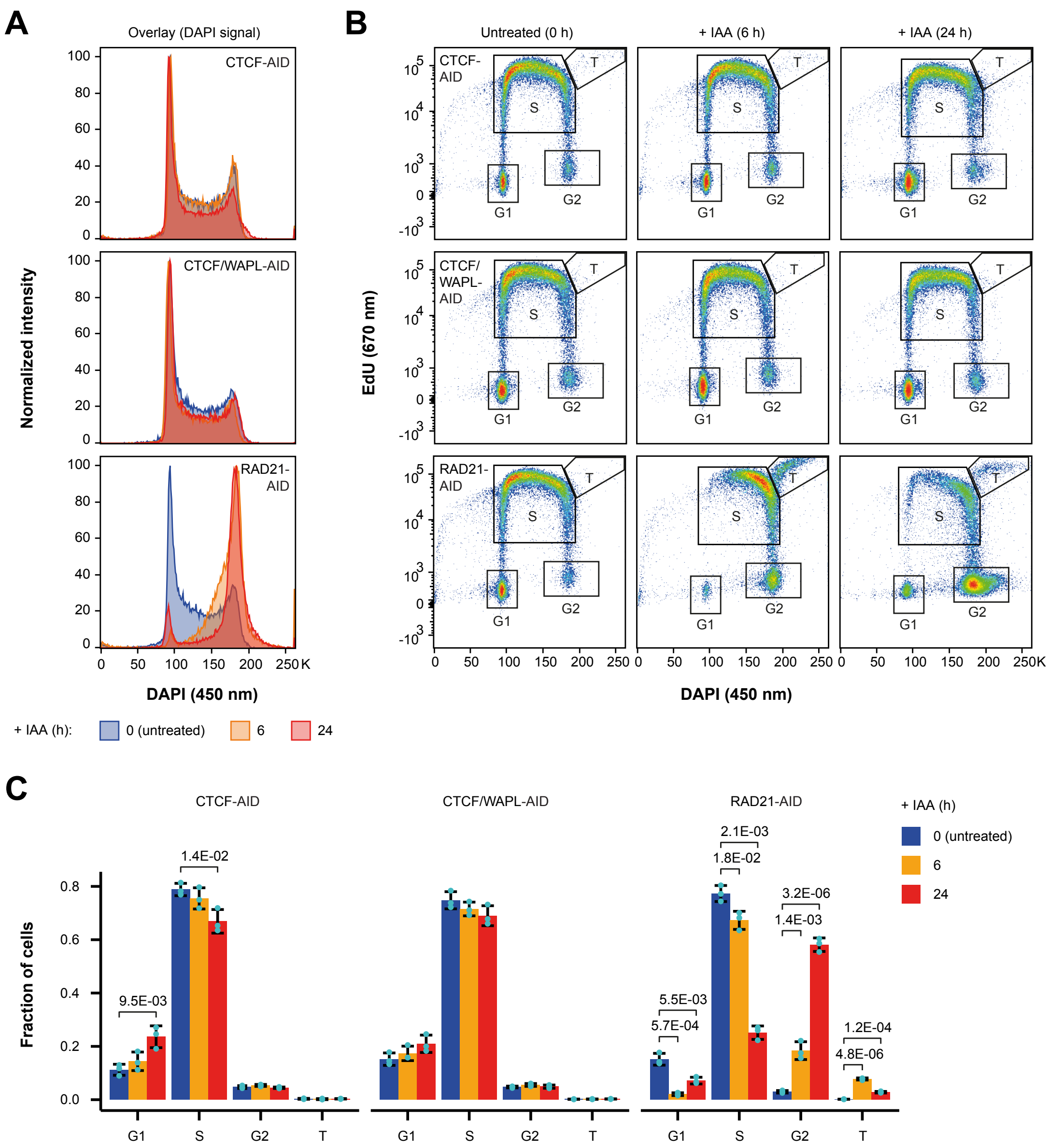

Fig S3. Effect of protein depletions on the cell cycle

(A) Density profiles of DNA content (DAPI signal) for a representative experiment in mESCs during a time course of IAA-mediated depletion of CTCF, RAD21 and CTCF/WAPL. **(B)** FACS plots showing EdU incorporation to measure the cycle phase of individual cells. All single cells are gated, and G1, S and G2 cells are counted. We included a gate for tertiary cells (T) that presumably reflect cells that undergo a second round of DNA replication without prior cell division. **(C)** Quantification of cycle phases based on DAPI/EdU FACS analysis for various times after CTCF, RAD21 and CTCF/WAPL depletion. Data are from three independent experiments. Mean values (bars), standard deviation (error bars) and individual replicates are visualized. Significant changes (two-sided t-test) are highlighted.

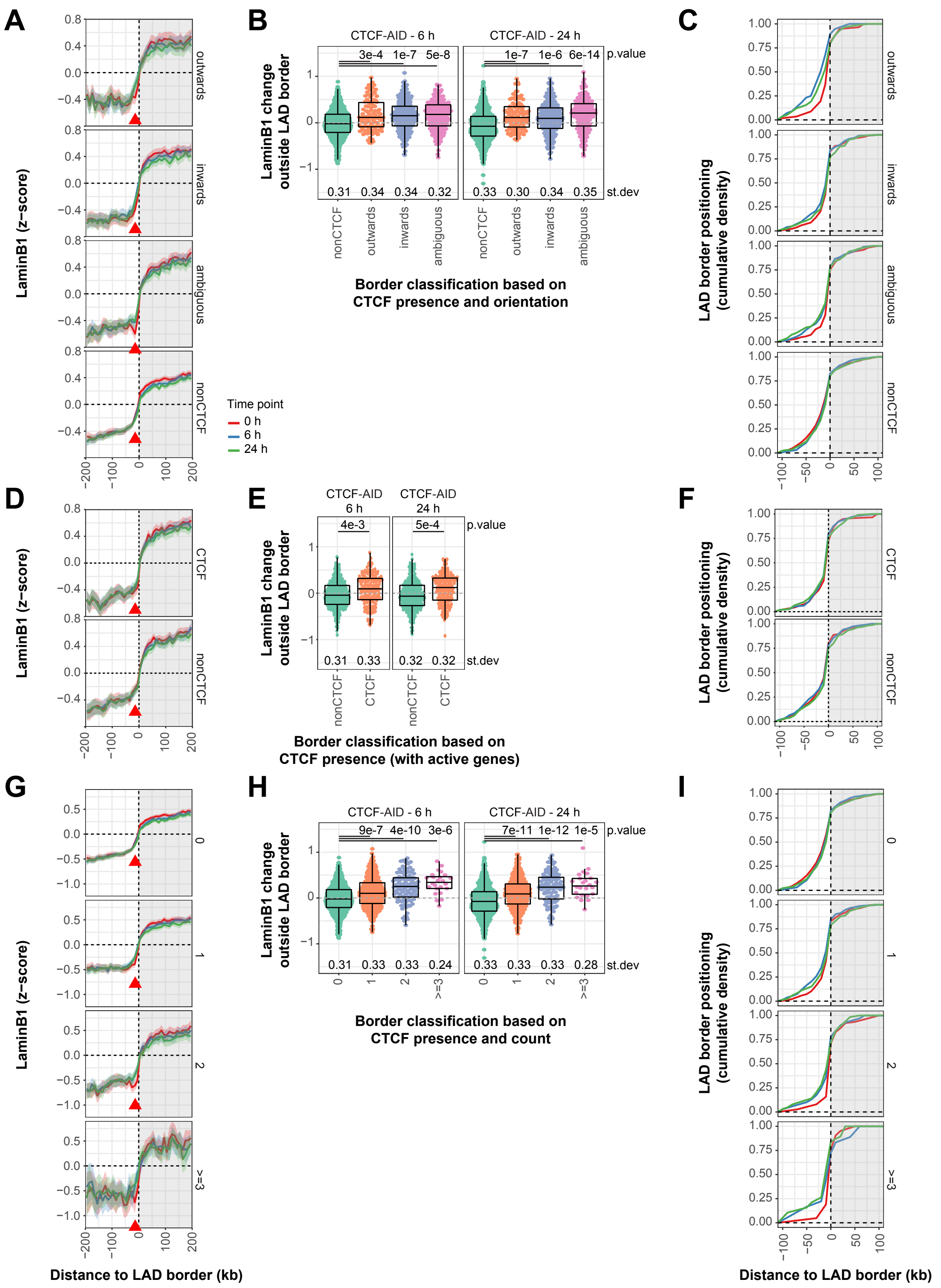

Fig S4. Further analysis of the effect of CTCF depletion on LAD border positioning

(A-C) Profiles similar to (Fig. 1E-G), showing average LaminB1 z-scores (A), LaminB1 changes outside LADs (B) and LAD border positioning (C) for LAD borders classified with CTCF motif orientation. **(D-F)** The effect of CTCF depletion at LAD borders close to active genes, classified by CTCF presence. Plots are similar to panels (A-C). **(G-I)** The effect of CTCF depletion at LAD borders classified by the number of CTCF sites within 20 kb. Plots are similar to panels (A-C).

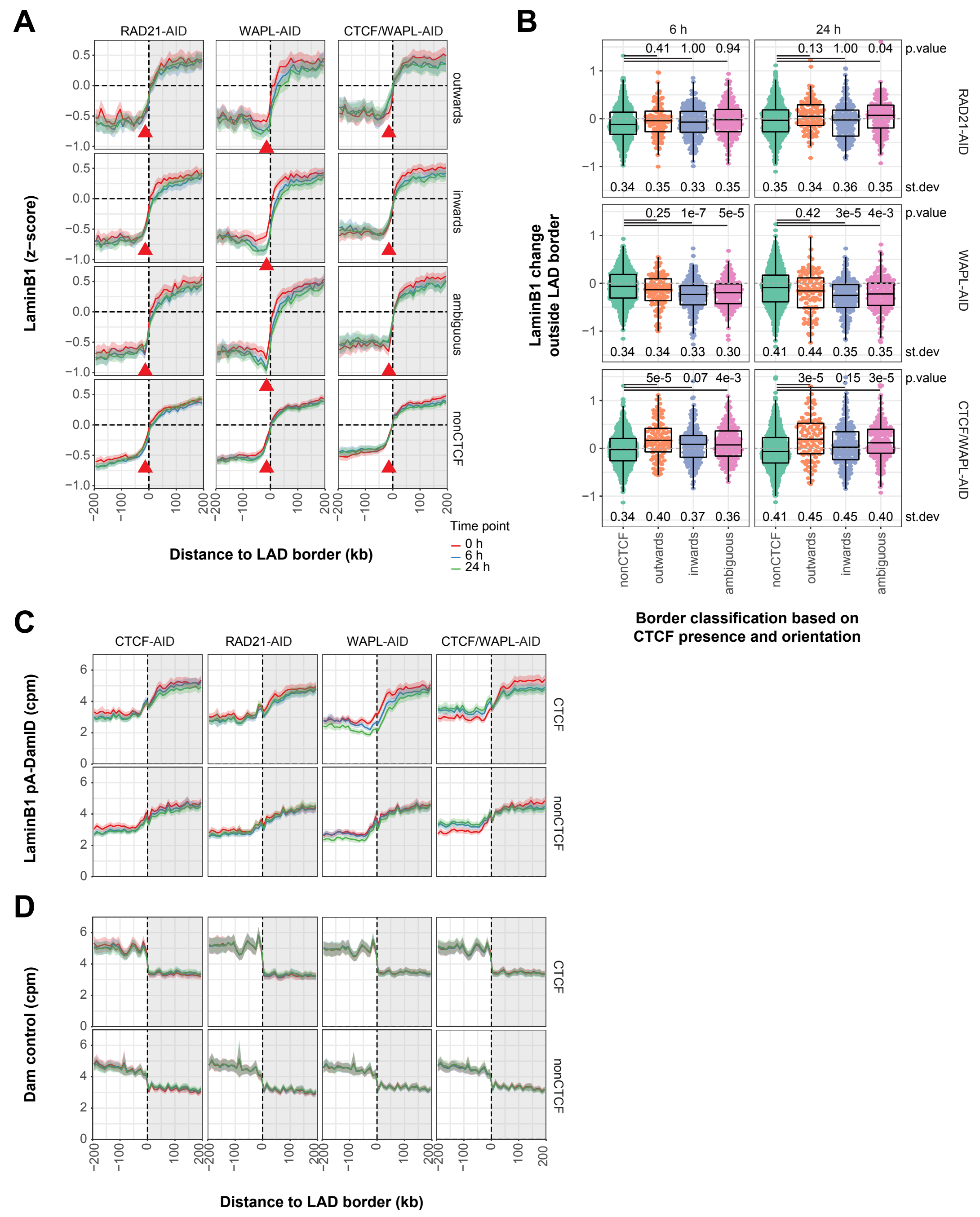

Fig S5. Perturbed NL interactions are limited for outwards oriented LAD borders and caused by changes in LaminB1 reads

(A-B) Profiles similar to (Fig. 2C-D), showing average LaminB1 z-scores for LAD borders classified with CTCF motif orientation (A) and the change in LaminB1 z-scores outside LAD borders for individual LAD borders (B). **(C-D)** Counts-per-million normalized (cpm) LaminB1 (C) and Dam-control (D) signals are shown around LAD borders.

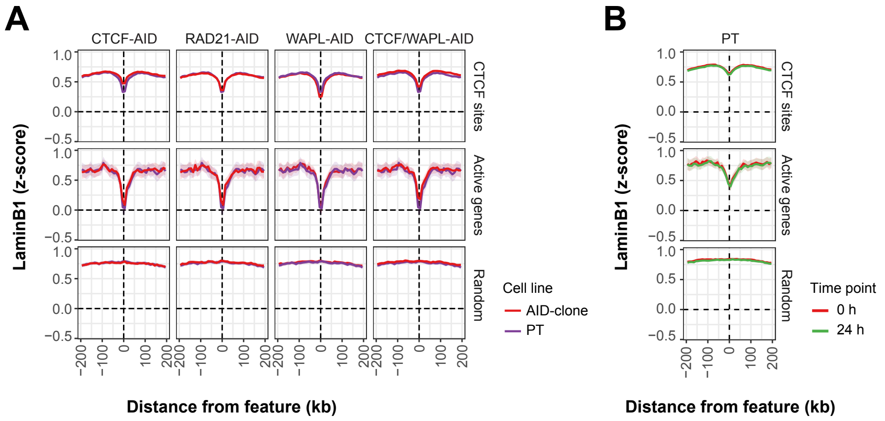

Fig S6. Protein depletion by AID-tagging induces a partial effect on CTCF detachment within LADs
(A) Overlay of the pA-DamID z-scores in the PT clone and the AID-tagged clones in untreated conditions (0 h). Data is the same as in (**Fig. 3C**). **(B)** Plot similar to (**Fig. 3C**), but instead showing the parental data described throughout the manuscript (generated with a new lot of LaminB1 antibody). The untreated data (0 h) is reused from (**Fig. 3A**).

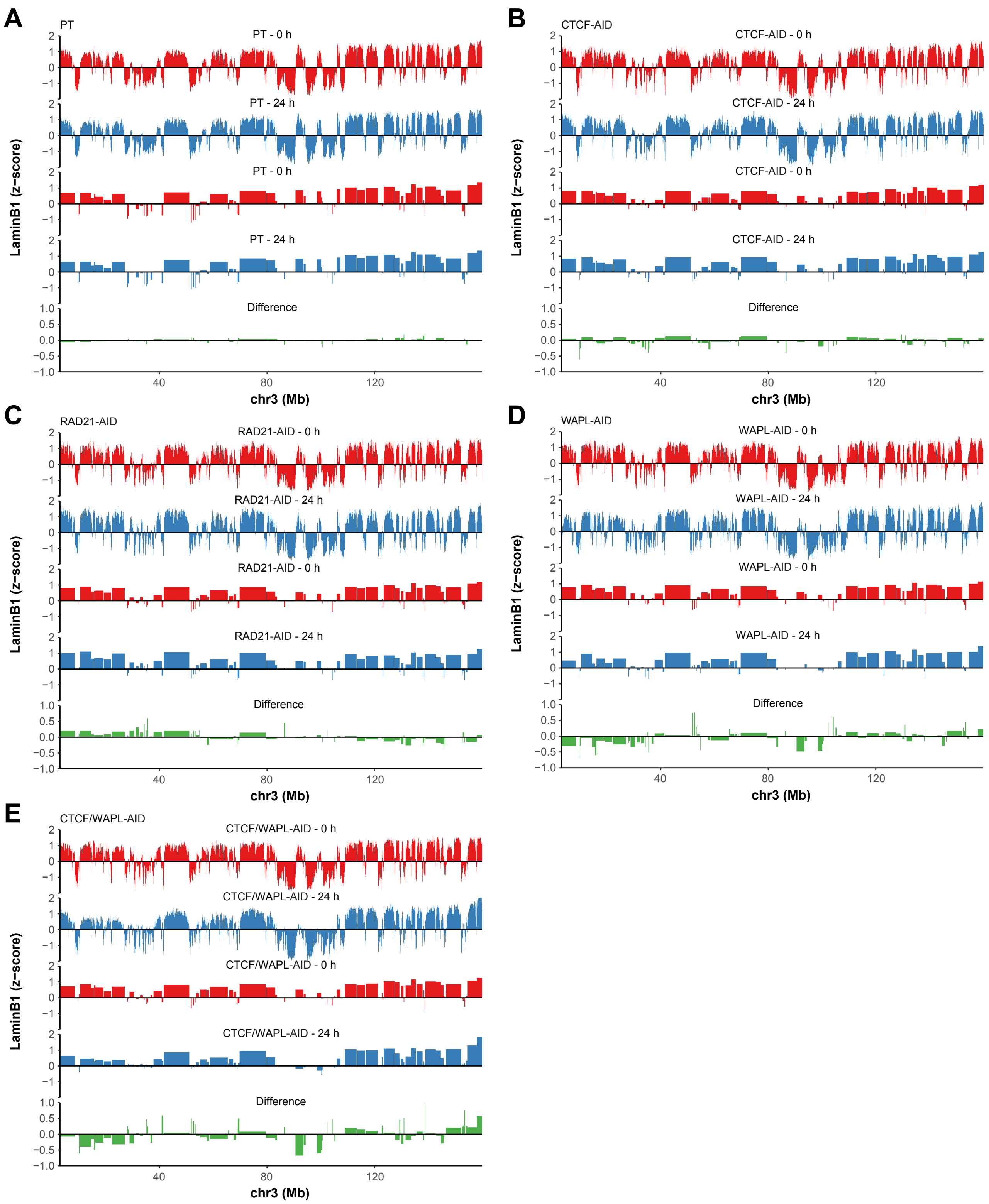

Fig S7. Quantification of LAD differences after CTCF depletion and cohesin perturbation (A-E) Overview of the quantification of NL interactions changes along a representative chromosome, for PT (A), CTCF (B), RAD21 (C), WAPL (D) and CTCF and WAPL (E) depletion experiments (tracks 1-2). A union set of LADs was created across all cell lines and conditions. The LAD score is defined as the average signal across all overlapping 10 kb bins (tracks 3-4). The difference in LAD score (track 5) was used to correlate with LAD features, such as LAD size, gene density and chromosomal positioning.

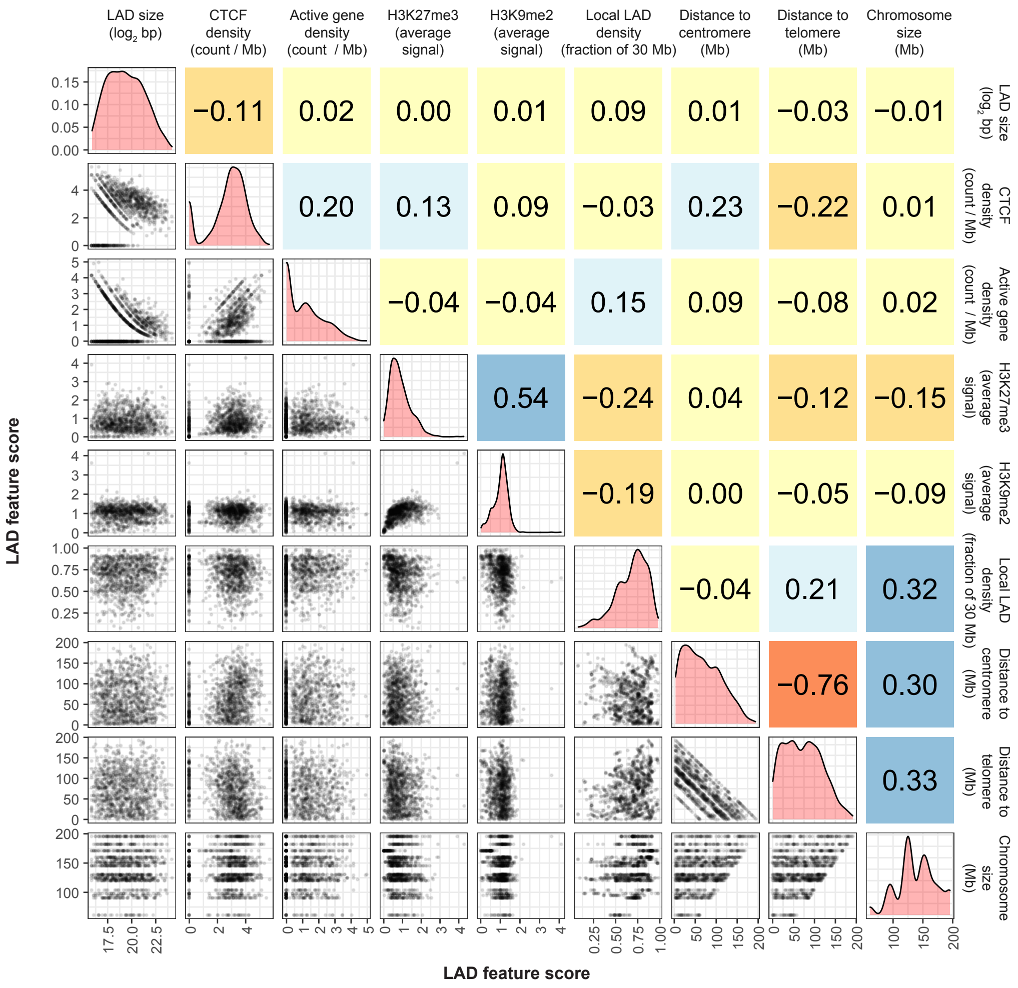

Fig S8. Correlation between LAD features

Correlation matrix between the LAD features, showing scatter plots (bottom-left panels), data distributions (diagonal panels) and Spearman correlations (top-right panels) for all comparisons.

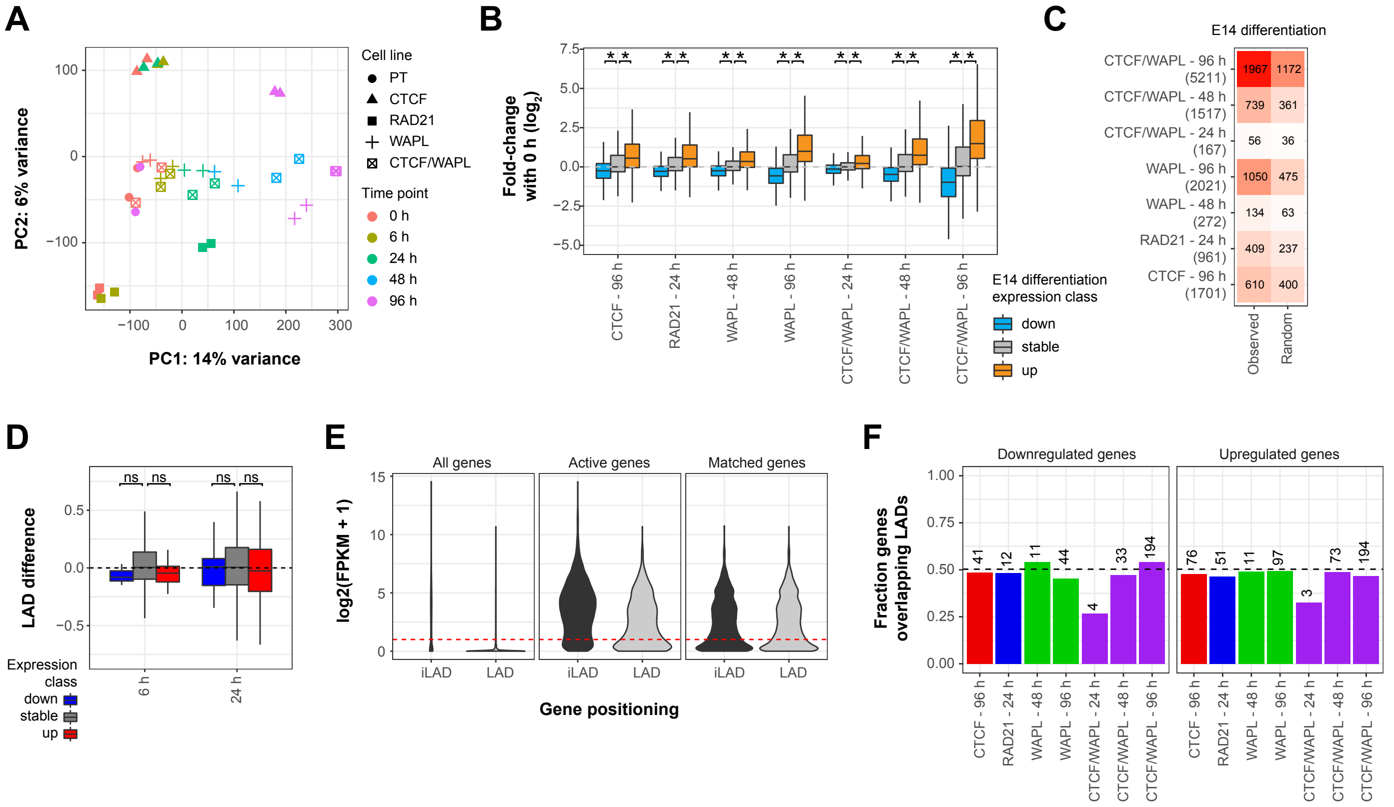

Fig S9. CTCF depletion and cohesin perturbation affect an overlapping gene set enriched for differentiation genes

(A) Principal component analysis (PCA) for all RNA-seq samples based on the top-5000 most variable genes. **(B)** Box plots showing expression fold changes ($\log_2$) relative to 0 h of protein depletion for differentially expressed genes during mESC to mNPC differentiation. Differentiation data is from ref [55]. Only time points with at least 50 differentially expressed genes are shown. Significance was tested for using a Wilcoxin test followed by Benjamini-Hochberg multiple testing correction. **(C)** Comparison between the observed overlap in differentially expressed genes between IAA-mediated protein depletion and mNPC differentiation, as described in (Fig. 5B). **(D)** Similar plot to (Fig. 5C), but instead showing LAD differences classified by nascent transcription data after IAA addition in WAPL-AID cells. **(E)** FPKM distributions for genes positioned in iLADs and LADs. Genes were filtered to be active in at least one condition (active genes) and a random matching set of iLAD genes was selected with similar expression to the LAD genes (matched genes). 10 random sets were taken for a robust comparison. **(F)** Fraction of differentially expressed genes positioned in LADs for using an equal-sized matched expression set of LAD and iLAD genes (E). A fraction of 0.5 indicates that LAD and iLAD genes are equally affected by protein depletions. The number denotes the number of differentially expressed LAD genes.

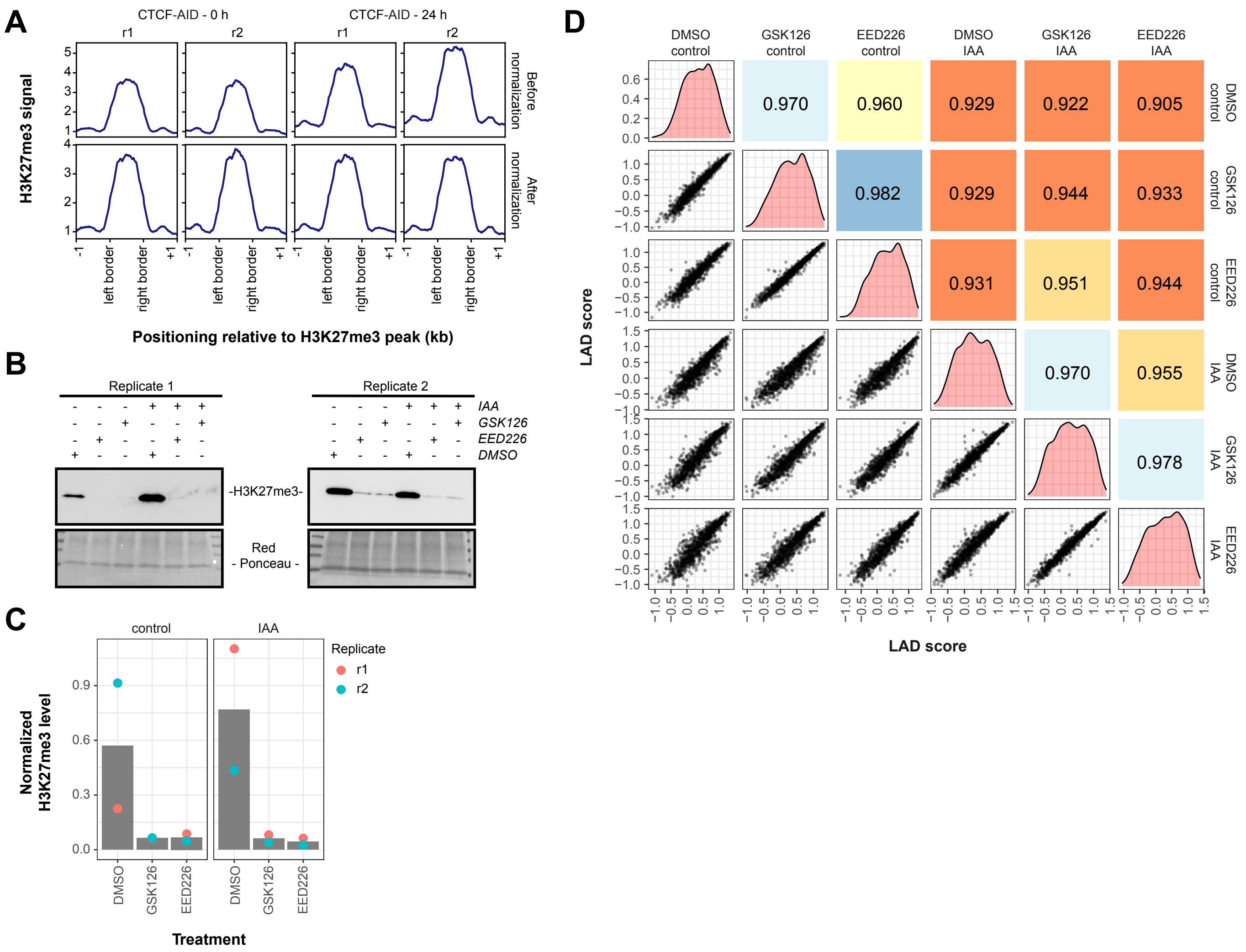

Fig S10. H3K27me3 depletion does not affect genome-wide NL interactions

(A) Overview of the calibration of the H3K27me3 ChIP-seq data (n = 2), illustrated by the average H3K27me3 signal around H3K27me3 peaks on the human genome hg19. mESC samples were mixed to contain 10% human HEK293T cells. The read normalization scaling factor for the human reads was also applied on the mm10 reads (see Methods). Human reads combined from all experiments were used to call the H3K27me3 peaks visualized here. **(B)** Western blot analysis of H3K27me3 levels in CTCF-AID mESCs treated for 3 days with the H3K27me3 inhibitors GSK126 (1 µM final concentration from 1 mM stock solution in DMSO) and EED226 (10 µM final concentration from 20 mM stock solution in DMSO) or a similar amount of DMSO as control, with and without 24 hours of IAA addition (cropped, n = 2). **(C)** Quantification of H3K27me3 levels in (B) compared to loaded protein (Ponceau staining). **(D)** Correlation matrix of LAD scores for the conditions listed in (B), showing scatter plots (bottom-left panels), data distributions (diagonal panels) and Pearson correlations (top-right panels) for all comparisons. Note that the variation upon IAA addition is larger than the variation induced by H3K27me3 depletion.
