# Supplementary material for: CTCF and cohesin promote focal detachment of DNA from the nuclear lamina
Source: Genome Biol. 2022 Sep 1;23:185. doi: 10.1186/s13059-022-02754-3 (PMC9438259; doi:10.1186/s13059-022-02754-3)
Supplement: Supplementary file 2 — Additional file 2: Table S1. List of datasets used. [file 13059_2022_2754_MOESM2_ESM.pdf]

Additional File 2: Table S1. List of datasets used.

Data type	Cell type	Target	Source	ID	Ref
DamID data to define LADs					
DamID	mESC	LaminB1	4D Nucleome; this work	4DNESYPMROEJ	77.
DamID	mNPC	LaminB1	This work	GSE183958	78.
DamID	H1	LaminB1	4D Nucleome; this work	4DNESXKBPZKQ	79.
DamID	Hap1	LaminB1	4D Nucleome; van Schaik, 2020	4DNESUK5H9Y8	80.
DamID	K562	LaminB1	4D Nucleome; van Schaik, 2020	4DNESTAJJM3X	81.
DamID	HCT116	LaminB1	4D Nucleome; van Schaik, 2020	4DNES24XA7U8	82.
CTCF ChIP-seq to determine LAD border enrichment					
ChIP	mESC	CTCF	Liu, 2021	GSM3992899	83.
ChIP	mNPC	CTCF	This work	GSE183958	78.
ChIP	H1	CTCF	ENCODE	ENCSR000BNH	84.
ChIP	Hap1	CTCF	Haarhuis, 2017	GSM2493878	85.
ChIPmentation	K562	CTCF	Schmidl, 2015	SRR2085872	86.
ChIP	HCT116	CTCF	ENCODE	ENCSR240PRQ	87.
pA-DamID data generated (after acute protein depletion)					
pA-DamID	mESC + various conditions	LaminB1	This work	GSE181693	88.
pA-DamID	Hap1	LaminB1	4D Nucleome; van Schaik, 2020	4DNESFWILAC9	89.
pA-DamID	K562	LaminB1	4D Nucleome; van Schaik, 2020	4DNESUMP6SS1	90.
pA-DamID	HCT116	LaminB1	4D Nucleome; van Schaik, 2020	4DNESWB729QB	91.
RNA-seq generated after acute protein depletion					
RNA-seq	mESC PT		Liu, 2021	GSE135180	92.
RNA-seq	CTCF-AID		This work	GSE181849	93.
RNA-seq	RAD21-AID		Liu, 2021	GSE135180	92.
RNA-seq	WAPL-AID		Liu, 2021	GSE135180	92.
RNA-seq	CTCF/WAPL-AID		This work	GSE181849	93.
ATAC-seq generated after acute protein depletion					
ATAC-seq	CTCF-AID		This work	GSE181846	94.
ATAC-seq	RAD21-AID		This work	GSE181846	94.
ChIP-seq generated after acute protein depletion					
ChIP	CTCF-AID	H3K27me3	This work	GSE181847	127.
mESC loop pairs					
Hi-C	Loop pairs		Bonev, 2017	GSE96107	95.
mESC epigenetic data sets					
ChIP	mESC	RAD21	Liu, 2021	GSM3992901	96.
ATAC	mESC		Tastemel, 2017	GSM2651155	97.
ChIP	mESC	H3K4me1	Joshi, 2015	GSM1856424	98.
ChIP	mESC	H3K4me3	Marks, 2012	GSM590112	99.
ChIP	mESC	H3K9me2	von Meyenn, 2016	GSM2051618	100.
ChIP	mESC	H3K27ac	Joshi, 2015	GSM1856426	101.
ChIP	mESC	H3K27me3	Højfeldt, 2018	GSM2779214	102.
ChIP	mESC	H3K36me3	Marks, 2012	GSM590120	103.
H1 epigenetic data sets					
ChIP	H1	RAD21	ENCODE	ENCSR000BLD	104.
DNAse	H1		ENCODE	ENCSR794OFW	105.
ChIP	H1	H3K4me1	ENCODE	ENCSR631RJR	106.
ChIP	H1	H3K4me3	ENCODE	ENCSR019SQX	107.
ChIP	H1	H3K9me3	ENCODE	ENCSR883AQJ	108.
ChIP	H1	H3K27ac	ENCODE	ENCSR000ANP	109.
ChIP	H1	H3K27me3	ENCODE	ENCSR216OGD	110.
ChIP	H1	H3K36me3	ENCODE	ENCSR476KTK	111.
HCT116 epigenetic data sets					
ChIP	HCT116	RAD21	ENCODE	ENCSR000BSB	112.
DNAse	HCT116		ENCODE	ENCSR000ENM	113.
ChIP	HCT116	H3K4me1	ENCODE	ENCSR161MXP	114.
ChIP	HCT116	H3K4me3	ENCODE	ENCSR333OPW	115.
ChIP	HCT116	H3K9me3	ENCODE	ENCSR179BUC	116.
ChIP	HCT116	H3K27ac	ENCODE	ENCSR000EUT	117.
ChIP	HCT116	H3K27me3	ENCODE	ENCSR810BDB	118.
ChIP	HCT116	H3K36me3	ENCODE	ENCSR091QXP	119.
RNA-seq in other cell types for active gene definition (FPKM > 1)					
RNA-seq	mNPC		Bonev, 2017	GSE96107	95.
RNA-seq	H1		ENCODE	ENCFF000FET, ENCFF000FEU, ENCFF000DJM, ENCFF000DJN, ENCFF565ZQD, ENCFF953ZDW, ENCFF589VNC, ENCFF608OLY, ENCFF247WDK, ENCFF350HDB, ENCFF199CUO, ENCFF687WLZ, ENCFF199CUO, ENCFF567PCA	120.
RNA-seq	K562		ENCODE	ENCFF001RED, ENCFF001REG, ENCFF001RWD, ENCFF001RVV, ENCFF001RWE, ENCFF001RWF, ENCFF001RDD, ENCFF001RDE, ENCFF000HFF, ENCFF000HFH	121.
RNA-seq	HCT116		ENCODE	ENCFF000DKT, ENCFF000DKW, ENCFF000DKV, ENCFF000DKX, ENCFF000DKY, ENCFF000DKU	122.
RNA-seq	HCT116		Kelso, 2017	GSM2719768, GSM2719769	123.
RNA-seq	HCT116		Dai, 2018	GSM2775145, GSM2775146	124.
RNA-seq	Hap1		Haarhuis, 2017	GSM2493886, GSM2493887, GSM2493888, GSM2493898, GSM2493899, GSM2493900	125.
RNA-seq	Hap1		Essletzbichler, 2014	SRX655511, SRX655512	126.
