# Supplementary material for: CTCF and cohesin promote focal detachment of DNA from the nuclear lamina
Source: Genome Biol. 2022 Sep 1;23:185. doi: 10.1186/s13059-022-02754-3 (PMC9438259; doi:10.1186/s13059-022-02754-3)
Supplement: Supplementary file 3 — Additional file 3. Review history [file 13059_2022_2754_MOESM3_ESM.docx]

**1st round**

**Reviewer 1**

van Schaik et al. present a manuscript where they explore the role of CTCF, cohesin and WAPL in shaping (and fine-tuning) genome-nuclear lamina contacts. The authors perform protein depletion experiments followed by pA-DamID to interrogate the effect (individually or in combination) of depleting these proteins. Even though their results indicate rather minor effects, in general affecting (some) LAD borders only, the paper is an interesting read and answers important pressing questions in the field. Below, I highlight some aspects in which the manuscript can be further improved.

1) The authors state multiple times that they observe "small differences" or "various small changes" in NL interactions upon e.g. CTCF depletion (see e.g. p.6, l. 31; p.7, l.12). These statements should be substantiated by providing the actual numbers. I.e, how many LADs (absolute numbers, and basepairs) are affected? And where in the genome are these changes located?

2) Even though the authors relate their results to the formation of "loops", they do not go much into how their results could (potentially) be linked to the now well-established mechanism of loop extrusion. As judged from e.g. fig 3C, it looks as if stalling of loop extrusion at CTCF binding sites has a role in focal detachment from the nuclear lamina. This point seems to be mentioned in the abstract (p. 2, l.8), but is not really picked up and discussed in more detail later in the manuscript. Thus, a deeper discussion on how loop extrusion is potentially involved should be provided.

3) Further, multiple authors [see e.g. Nuebler et al 2018] have previously reported an inverse relationship between loop extrusion and (A/B)-compartment strength. Thus, upon depletion of loop extrusion (cohesin), compartments seem to strengthen, including a sharpening of their transitions. There could be interesting links between these findings and the LAD border detachments reported here. The authors should complement their results with an analysis or discussion of how LAD border detachment could be related to A/B compartment changes upon cohesin/CTCF/WAPL depletion.

4) Finally, the authors treat each CTCF site independently. There are recent indications that clusters of multiple CTCF sites at loop borders are relevant for the stability of loops, such that borders with more CTCF sites are more stable than those harboring fewer CTCF sites [see e.g. Li-Hsin et al. 2020]. The authors should investigate whether there is a relationship between the number of consecutive CTCF sites and the effect of their depletions on lamina detachment.

Nuebler, Johannes, et al. "Chromatin organization by an interplay of loop extrusion and compartmental segregation." Proceedings of the National Academy of Sciences 115.29 (2018): E6697-E6706.

Chang, Li-Hsin, Sourav Ghosh, and Daan Noordermeer. "TADs and their borders: free movement or building a wall?." Journal of molecular biology 432.3 (2020): 643-652.

**Reviewer 2**

"CTCF and cohesin promote focal detachment of DNA from the nuclear lamina" by Schaik et al., attempts to achieve resolution of the many contradictions in the literature on the question of the role of CTCF with respect to LADs by using the AID-degron system to rapidly degrade CTCF and WAPL and then using the recently developed pA-DamID system to map nuclear lamina contacts. I was quite hopeful when I agreed to accept this reviewer assignment that they might have succeeded considering the track record of the van Steensel lab. Indeed they did not disappoint in generating a lot of quality data, but after reading the manuscript and reviewing the data I would say the verdict is still out on the role of CTCF and that for publication this needs both more analysis of the data and considerable rephrasing of conclusions.

What is clear in the data is that there is a CTCF peak that occurs on the non-LAD side and close to the LAD border. It is variable in its sharpness but can taper off from 10 to 25 kb away from the LAD border. They state that there was no enrichment between constitutive and facultative LADs, but I do not see data for this. Then it is clear that there is no difference depending on orientation of the CTCF motif nor any consistency or particular enrichment of active genes between the areas adjacent to LAD borders that have CTCF versus those that do not. In panel B they state that the percentage of LADs with CTCF within 20kb ranged between 42 and 62% between the different cell types, yet the numbers on the bar graphs would not seem to match this by eye at least from the scale: perhaps they need to be replotted or at least to add the percentages next to each bar or perhaps my understsanding is off and there needs to be more description in the legend.

Many other parameters were investigated to see what else is at the subset of LAD borders defined by this cutoff, with many parameters previously shown confirmed and nothing new of particular interest found. They then exclude borders near active genes from their remaining analyses, which is argued to be preventing effects from transcription affecting analysis, but it seems that both analyses should be done in case someting interesting is observed in the active set since both the distal tethering of the lamina and CTCF-mediated loops could theoretically mechanically help stabilize genes for more efficient transcription. Indeed such would make sense for the original simple view of the lamina as a scaffold, yet, while we now acknowledge the lamina to be much more, people in the field sometimes neglect this function (that nonetheless is still there) in favor of hypotheses around epigenetic factors. I might have missed it, but I don't think anyone ever tested nuclear mechanics in the various CTCF depeleted systems. Note, I am not asking for this experiment to be done for this paper though having the system in place it might be worth collaborating with a mechanics person to do this: here, though, I would like to know if there might be some correlations from separately analyzing the data specifically for the transcriptionally active sites as this only involves using the algorithms already developed on a different subset of the data.

In any event, next they used the degron system to deplete CTCF. While I agree that this is often an elegant system that has in the past shown for a number of proteins that effects reported for longer knockdowns were indirect quaternary effects, I was disturbed at the fact that for both CTCF and the subsequent WAPL constructs in Figure S2A there appears to be a huge reduction in the levels at the 0 h timepoint and this could just as easily have yielded such quaternary effects. This data should not be in supplemental but be in the main figure and this potential limitation should be made very clear in the text.

The use of the pA-DamID is also an elegant contribution that one would have expected to help to finally answer the question of what CTCF does with relation to LADs. However, there was very little change in the DamID traces with only a very small subset of LADs showing changes. While I certainly wanted to see the individual trace examples that were given in Figure 1D and the global data that was given in Figure 1E and F, I also would have liked to see percentage data of the number of CTCF proximal LADs with changes and the number of non-CTCF proximal LADs with changes. Based on the DamID traces shown I would guess these percentages to be very small so that the very minor shifts in the global curves likely reflect larger changes at very few loci rather than a cumulative minor shift and tests should be done on the data and more traces added that can address this point.

The one more striking shift observed in panel E is a gain in nuclear lamina association in the adjacent inter-LAD region upon CTCF depletion. This is interpreted as meaning that CTCF contributes to the sharpening of LAD borders, but there are many other possible interpretations of the data, including mechanical ones. It could be simply that the CTCF-based loops help hold the DNA more internally in the nucleus so that it is harder to establish lamina interactions with a larger region that has affinity or many other possibiltiies including those involving epigenetics. Thus, such conclusions that seem to be trying to get the reader to just accept their particular interpretation need to be removed from the text unless additional data is shown that discounts the other hypotheses.

They next test the role of cohesin by its indirect stabilization or depletion with AID-WAPL and AID-RAD21. Again there is the issue that it is unclear what percentage of the different types of LAD borders are affected and this should be added, but there were also some slightly larger changes in the global profiles. Nonetheless, there were also more significant changes in the body of the LADs themselves which suggests that just as depletion of lamins can yield massive changes in the nucleus to generate lots of indirect artefacts, it is also quite possible that altering cohesin even in the short term would have similar indirect effects. Despite the textual statements that cohesin is playing a clear role, to me the traces of the combined CTCF and WAPL depletion look extremely similar to that of CTCF alone back in Figure 1. I would really like to see these traces overlaid as to me this would suggest that the predominant effect is through CTCF depletion and that the effects seen when affecting cohesin alone are likely indirect.

The narrative continues with the assumption that CTCF binding actively drives the detachment of a never clearly stated amount of inter-LADs chromatin as a directed function and next I was a little confused with the terminology describing the CTCF sites being inside LADs: since these sites are detached from the nuclear lamina then since the definition of a LAD is being at the lamina I don't think they should be called as part of LADs. Do the authors mean facultative LADs that are changing? or that they are only weak breaks from the LAD in traces because only a small subset of the pooled population has them broken away from the nuclear lamina? Regardless, it was also unclear whether the actively transcribed genes that are broken away also from LADs also have CTCF, but they show no effect of depletion on the actively transcribed gene regions broken away from LADs whereas the CTCF islands that are not actively transcribed genes in regions that have broken away from LADs were affected. This all needs to be cleared up and described better, but, again, from the standpoint of overintepreting, I would think it equally likely that the CTCF and transcription likely prevent the spread of epigenetic marks through these regions and/or sterically block attachment to the lamina and when the CTCF goes away this no longer happens and it gets marked and bound. So to address this properly would be to NOT drop further study of the transcriptionally active sites as they did, but rather test what happens when they knock off the transcription factors and block transcription and see if that then results in those regions associating with the lamina +/- co-depletion of CTCF - that is if the CTCF is actually also in those sites. Then the other thing would be to do the ChIP-Seq for the epigenetic marks and see if they change with the CTCF depletion in the non-transcriptionally active CTCF islands. Note also that I was also not clear in this section on whether the epigenetic data were all previous data? I assume so because there is no distinction about changes due to CTCF depletion and no epigenetic ChIP-Seq is described in the methods; however, it does seem like it might have been interesting to check if these change with the CTCF and other depletions generally for other experiments as well and it seems also that not doing so implies that these are not changing when they may well be.

Re the need for new data, it was not clear what data was being used to calculate loop anchors, but this seems too like something where to do it properly one should get Hi-C data from the same +/- CTCF system used. In general, it was possible to find some answers to questions about what data was used by sifting through the methods section, but I think this should be being stated in the text throughout what data was used and likewise what external datasets - note this latter is relegated entirely to the supplementary information, but this is information that belongs in the results section. Sifting through the methods also explained with how LADs were called why these non-LAD regions are being called as within LADs, but I still think it would be more clear to reduce the bin sizes and to not call them LADs in the first place. So there needs throughout the text to be more orientation of the reader as to what data is actually being used and what is being compared so that they do not have to keep checking the supplementary data, figure legends, and methods to still sometimes only guess at this.

The correlations in Figure 4 strike me as not being as great as the text would imply. I can understand why Spearman and not Pearson was used here, but even for Spearman to my understanding a strong correlation is at 1 and their plot seems to only go to about 0.25 according to their gradient key. Yet they state that there are significant correlations. What is potentially interesting is the way some parameters switch from leaning towards correlating to anticorrelating between different depletions, but presented as it is this data does not give the confidence of interpretation that the text does. Perhaps their color system is not as good as just putting the numbers in the individual boxes and it would be better to remove the color gradient and just shade the boxes with significance and then put the actual numbers inside, but from how the data is presented I am hesitant to embrace the textual interpretations here. At the same time, if reasonable numbers were clearly given then I would say this is a place where there are some more interesting relationships where we could say the depletion more affects a particular chromosome region etc. At the same time, this data also needs to be elaborated and expanded on if this is the case because for example for the telomere suggested correlation one needs to also see the distribution of both LADs and CTCF binding sites at telomeres to judge whether this might just be an effect due to greater densities of CTCF binding here or something reflecting a unique function here. This would also require knowing what percentage of sites in these regions changed compared to other regions controlled for density and other factors. Also at the same time, a correlation like the small LAD with CTCF versus the big LADs with WAPL could just reflect the possible general genome-wide pleiotropic effects of messing with cohesin for why larger LADs tend to be affected and not necessarily something reflecting a specific functionality.

Next the transcription Figure 5 is straigtforward but just negative data and the epigenetics Figure 6 would have been better to do ChIP-Seq +/- the depletions than using inhibitors. Moreover, there could easily be combinatorial epigenetic signatures that would only be identified by looking at multiple marks in the various depletion lines. Thus, to conclude that there is no interplay with even just the one is perhaps premature, but the implication would appear to be that epigenetics in general is not likely interplaying and since more was not tested there should be a lot more qualification of these conclusions. This gets to the biggest issue, I highlighted a number of individual points above where it would be worth doing a bit more analysis of the existing datasets and also perhaps do a bit of ChIP-Seq on the lines generated for this study, but with the data that hs been presented there really needs to be a significant cut back in the strength of conclusions made throughout. In particular, the conclusions as stated in the abstract are that CTCF is significantly at LAD borders, but its depletion doesn't change much, yet nonetheless it is important for "reinforcing" LAD borders and is important to "mediate fine-tuning of NL interactions". The data is clear that some fraction of LADs change when CTCF or cohesin are depleted, but whether the function is to reinforce is never actually tested in any meaningful way and the use of the term fine-tuning is also not supported with any actual data. Just that some small fraction has an effect does not mean the role is to fine tune something. What are the specifically so-regulated genes and functional consequences of their role if they are really fine tuning? If one was looking at a functional output such as transcription, then this term would suggest that the transcription factors etc are around but some aspect of the CTCF interaction with the LAD gives an additional bit of regulation to the system such as making the on/off regulation of the gene tighter or that normally CTCF goes off at a certain time to affect the temporal dynamics of the gene's regulation with a particular defect that is apparent that could lead to pathology if it did not have this extra bit of fine-tuning regulation. However, no experiments show anything like this for any kind of functional output and I am not certain what would be useful to test here since they dropped investigating transcription early on. So the conclusions strike me as massively overstated.

Another general question I have is where did the 20kb cutoff come from and how valid is it? It was stated that the peak was at 10kb, but with the accuracy of mapping LAD boundaries this could be even closer and even 10kb could be physically far from the nuclear envelope. We still don't know in what form DNA interacts with the nuclear lamina, but at 200 bp per nucleosome and 10-11 nm wide in a 10 nm fiber, even without linker histones at lower density, this would be 100 x 10 nm = 1 µm which is 1/5 the diameter of the average mammalian nucleus. Even if assuming that 30 nm fibers are interacting and using one of the tightest packing models at a 10x further shortening, this would still be far enough away that it is hard to imagine CTCF binding sites at this distance having a huge impact on the specific binding of the LAD boundaries. So just changing these parameters could potentially find correlations that were not apparent with the current analysis. It also would be beneficial to know numbers for what percentage of a particular subgroup of CTCF-associated LADs (versus non-associated) does something throughout the study. For example, what percentage of the LADs in each category are facultative vs constitutive and how many of the facultative are enriched for developmentally regulated genes. We get in Supplemental Figure S5 a statement that CTCF depletion and cohesin perturbation affect overlapping sets of differentiation genes, but nowhere is this data presented in a way that give the reader a sense of whether this is 90% of the genes changing in differentiation having some CTCF/cohesin connection or regulation or if it is 5% of the genes changing in differentiation or if CTCF/cohesin is enriched at developmentally changing genes since there could be an extra player in the regulation at this particular gene subset. It seems that after spending so much time and effort in making these elegant depletion systems and generating so much data looking at LAD changes that they should be asking these questions with the data.

**Reviewer 3**

CTCF and cohesin are major players in A-compartment genome organization and it has been long known that CTCF is enriched at LAD borders and, more recently, in local detached euchromatic regions within LADs. What has remained uncertain is the role that CTCF plays in the organization of LADs, if any. In this manuscript, van Schaik et al describe a series of acute depletion experiments to determine how CTCF and cohesin regulate LAD organization and borders. In addition, van Schaik et al investigate the role that H3K27me3 plays in CTCF regulated genome interactions and in LAD contacts with the nuclear lamina. Overall this is an intriguing set of studies that helps to clarify and demonstrate the LAD organization is independent of A-compartment organizing principles. There are some concerns with experimental set-up and analyses (outlined below) which dampen enthusiasm for this work as-is a little. Nonetheless, these studies are a valuable addition to the field and a necessary step forward in our understanding of LAD organization.

1 A general comment that applies to all the experiments outlined: given the perturbations, there should be an analysis of the cell cycle status and cell doubling during experimental manipulation time-frames. If the cycle is slowed or halted, in what cell cycle stage? If the cells arrest in G1/S/G2, this could mean that the perturbations do not affect the organization of established LADS, but would say nothing about whether reorganization requires these factors/chromatin modification (in the case of H3K27me3).

2. Data on LADs with active genes within 10 kb or the LAD border were excluded from the analyses. I am puzzled by this. The authors state this was to avoid "confounding factors", but this might be important to understanding LAD organization. I would like to see the parsed out data in a supplemental figure. Does it deviate from the filtered LAD border data? If so, how/why? It is unclear to me what confounding factors could precludes this inclusion or at least separate analyses in supplemental data. The two features are not unrelated, after all.

3. Looking at the western blots for the CTCF-AID, WAPL-AID, RAD21-AID< for both CTCF And WAPL-AID, it appears that CTCF and WAPL levels are *already* compromised prior to auxin mediated degradation. This could mean that the cells are already compromised. All data should have the parental control (not just 0 hours) as a baseline control. One concern is that the cells have compensated (in some way) for a depleted level of these factors thus obscuring some potentially important roles.

4. "Furthermore, the effects of RAD21 and WAPL on LAD-wide NL interactions both depend on the chromosomal position (i.e., distance to telomere and centromere), but in opposite orientation. This effect appears roughly linear with distance to the telomeres and is present across all chromosomes (Fig 4E). Chromatin nearby telomeres is enriched at the NL in early G1 cells and slowly loses interactions with the NL in interphase [33]. Possibly stabilized cohesin somehow prevents this normal maturation of NL interactions"

This once again highlights the need for a robust cell cycle analysis of the perturbed cells. This could be reflecting alterations in the cell cycle itself.

5. "Towards this goal we calculated mean pA-DamID scores on a consensus set of LADs across all conditions, and then determined the changes in scores in each depletion experiment (see Methods, Fig S4)."

I am concerned about the validity of this approach given that pA-DamID (DamID generally) is not known a quantitative measure of degree of association (likely there is some, but this has not been calibrated to be so). In order to discern the validity of this approach, parental cells need to be evaluated the same way (with each other between replicates and against the amalgam LAD maps). This is also a concern since the degree of depletion in the untreated cells varies (especially since some are starting with lower protein levels to begin with in the genetically engineered lines).

6. In the discussion regarding H3K27me3, it is suggested that H3K9me2 might be sufficient for LAD targeting, but this contradicts the findings that H3K9me2 alone is insufficient -as evidenced by H3K9me2 enriched chromocenters in mouse cells. Perhaps some of the findings can be explained by redundancies in organization? Also, the role of active transcription—either at LAD borders or generally may paly a role in this process.

7. The overall discussion of H3k27me3 in LAD organization is excellent, however given the overlap of CTCF with H3K27me3 and the role that LADs/H3k27me3 play in facultative gene regulation, is it possible that in ESC some of these findings are due to bi-valent or semi-poised genes? I think further discussion of the nature of gene regulation in ESC versus other cell types is warranted. In addition, the make-up of the lamin proteins and the nuclear envelope varies between cell types, notably the lamins themselves. (ESC having no lamin A, but instead reliant on LBR—Solovei 2013).

**Authors’ response**

**Reviewer 1**

van Schaik et al. present a manuscript where they explore the role of CTCF, cohesin and WAPL in shaping (and fine-tuning) genome-nuclear lamina contacts. The authors perform protein depletion experiments followed by pA-DamID to interrogate the effect (individually or in combination) of depleting these proteins. Even though their results indicate rather minor effects, in general affecting (some) LAD borders only, the paper is an interesting read and answers important pressing questions in the field. Below, I highlight some aspects in which the manuscript can be further improved.

We thank the reviewer for the helpful comments.

1) The authors state multiple times that they observe "small differences" or "various small changes" in NL interactions upon e.g. CTCF depletion (see e.g. p.6, l. 31; p.7, l.12). These statements should be substantiated by providing the actual numbers. I.e, how many LADs (absolute numbers, and basepairs) are affected? And where in the genome are these changes located?

We have tried to substantiate our statements by including additional figure panels (Fig. 1F, 2D, 4A-B, 5B) and providing actual numbers in the text (l. 197- 200, 256, 330). We agree that this conveys the data better.

2) Even though the authors relate their results to the formation of "loops", they do not go much into how their results could (potentially) be linked to the now well- established mechanism of loop extrusion. As judged from e.g. fig 3C, it looks as if stalling of loop extrusion at CTCF binding sites has a role in focal detachment from the nuclear lamina. This point seems to be mentioned in the abstract (p. 2, l.8), but is not really picked up and discussed in more detail later in the manuscript. Thus, a deeper discussion on how loop extrusion is potentially involved should be provided.

We have further explored this. Re-analysis of our data (including additional control datasets) indicates that the detachment is more pronounced when it involves looping with a second CTCF site that is located outside a LAD (revised Fig. 3G). Intuitively this makes sense: this loop, with one end located in the nuclear interior, may pull the LAD-based CTCF site away from the lamina. We have included a discussion on the relation between looping and focal detachment (l. 448-451).

3) Further, multiple authors [see e.g. Nuebler et al 2018] have previously reported an inverse relationship between loop extrusion and (A/B)-compartment strength. Thus, upon depletion of loop extrusion (cohesin), compartments seem to strengthen, including a sharpening of their transitions. There could be interesting links between these findings and the LAD border detachments reported here. The authors should complement their results with an analysis or discussion of how LAD border detachment could be related to A/B compartment changes upon cohesin/CTCF/WAPL depletion.

We agree that this is an important comparison to make. This comparison is included in a separate manuscript (available on bioRxiv: https://doi.org/10.1101/2021.08.27.457977), which shows that compartments are strongly affected upon WAPL and CTCF/WAPL depletion while LADs are mostly unperturbed. We now refer to this observation in the discussion (l. 484-487).

4) Finally, the authors treat each CTCF site independently. There are recent indications that clusters of multiple CTCF sites at loop borders are relevant for the stability of loops, such that borders with more CTCF sites are more stable than those harboring fewer CTCF sites [see e.g. Li-Hsin et al. 2020]. The authors should investigate whether there is a relationship between the number of consecutive CTCF sites and the effect of their depletions on lamina detachment.

This is a very good suggestion. We have included this analysis (Fig. 1F, S4G-H). We indeed observe that LAD borders with multiple CTCF sites are more strongly affected by CTCF depletion. This is in accordance with the model that multiple CTCF sites make stronger borders.

Nuebler, Johannes, et al. "Chromatin organization by an interplay of loop extrusion and compartmental segregation." Proceedings of the National Academy of Sciences 115.29 (2018): E6697-E6706.

Chang, Li-Hsin, Sourav Ghosh, and Daan Noordermeer. "TADs and their borders: free movement or building a wall?." Journal of molecular biology 432.3 (2020): 643- 652.

**Reviewer 2**

“CTCF and cohesin promote focal detachment of DNA from the nuclear lamina" by Schaik et al., attempts to achieve resolution of the many contradictions in the literature on the question of the role of CTCF with respect to LADs by using the AID- degron system to rapidly degrade CTCF and WAPL and then using the recently developed pA-DamID system to map nuclear lamina contacts. I was quite hopeful when I agreed to accept this reviewer assignment that they might have succeeded considering the track record of the van Steensel lab. Indeed they did not disappoint in generating a lot of quality data, but after reading the manuscript and reviewing the data I would say the verdict is still out on the role of CTCF and that for publication this needs both more analysis of the data and considerable rephrasing of conclusions.

We thank the reviewer for the constructive and very detailed comments. These have been helpful in improving the manuscript.

What is clear in the data is that there is a CTCF peak that occurs on the non-LAD side and close to the LAD border. It is variable in its sharpness but can taper off from 10 to 25 kb away from the LAD border. They state that there was no enrichment between constitutive and facultative LADs, but I do not see data for this. Then it is clear that there is no difference depending on orientation of the CTCF motif nor any consistency or particular enrichment of active genes between the areas adjacent to LAD borders that have CTCF versus those that do not. In panel B they state that the percentage of LADs with CTCF within 20kb ranged between 42 and 62% between the different cell types, yet the numbers on the bar graphs would not seem to match this by eye at least from the scale: perhaps they need to be replotted or at least to add the percentages next to each bar or perhaps my understsanding is off and there needs to be more description in the legend.

The reviewer is correct that we compared “shared and unique LAD borders”, which are defined differently from constitutive and facultative LADs. We have clarified the text (l. 128-131) and added more numbers to the figures to address the comments above (Fig. 1B, S1C, S2G).

Many other parameters were investigated to see what else is at the subset of LAD borders defined by this cutoff, with many parameters previously shown confirmed and nothing new of particular interest found. They then exclude borders near active genes from their remaining analyses, which is argued to be preventing effects from transcription affecting analysis, but it seems that both analyses should be done in case someting interesting is observed in the active set since both the distal tethering of the lamina and CTCF-mediated loops could theoretically mechanically help stabilize genes for more efficient transcription. Indeed such would make sense for the original simple view of the lamina as a scaffold, yet, while we now acknowledge the lamina to be much more, people in the field sometimes neglect this function (that nonetheless is still there) in favor of hypotheses around epigenetic factors. I might have missed it, but I don't think anyone ever tested nuclear mechanics in the various CTCF depeleted systems. Note, I am not asking for this experiment to be done for this paper though having the system in place it might be worth collaborating with a mechanics person to do this: here, though, I would like to know if there might be some correlations from separately analyzing the data specifically for the transcriptionally active sites as this only involves using the algorithms already developed on a different subset of the data.

The reviewer suggests to include LAD borders near active transcription. Because transcription is known to induce detachment from the nuclear lamina, we reasoned that it would be better to remove the effect of transcription to adequately study the direct role of CTCF on LAD border positioning. However, we agree that transcription and CTCF are not unrelated, and that we should include the combination to cover the full story. We have now clarified our reasoning (l. 146) and included a separate analysis of LAD borders near active transcription in Fig. S4D-F. Here, we observe the same loss in local detachment outside LAD borders, but do not find an effect on LAD border positioning.

In any event, next they used the degron system to deplete CTCF. While I agree that this is often an elegant system that has in the past shown for a number of proteins that effects reported for longer knockdowns were indirect quaternary effects, I was disturbed at the fact that for both CTCF and the subsequent WAPL constructs in Figure S2A there appears to be a huge reduction in the levels at the 0 h timepoint and this could just as easily have yielded such quaternary effects. This data should not be in supplemental but be in the main figure and this potential limitation should be made very clear in the text.

We agree that we should have emphasized better that CTCF and WAPL already have strongly depleted protein levels prior to auxin-mediated degradation. We have added this to the main text (l. 169, 228). However, we believe that it is better to leave the panel in the supplementary material. The figure shows protein levels of all depleted proteins in a single blot and adding this to figure 1 will complicate the narrative. If the reviewer believes it is crucial to include the panel in the main figures, we are happy to adjust this.

We now also look into the effect of the reduced protein levels on CTCF detachment (Fig. S6A). Although the biggest effect is observed upon depletion of the remaining protein by addition of auxin (see Fig. 3C), we do observe that the initial protein depletion of CTCF and WAPL in the absence of auxin also has an effect.

The use of the pA-DamID is also an elegant contribution that one would have expected to help to finally answer the question of what CTCF does with relation to LADs. However, there was very little change in the DamID traces with only a very small subset of LADs showing changes. While I certainly wanted to see the individual trace examples that were given in Figure 1D and the global data that was given in Figure 1E and F, I also would have liked to see percentage data of the number of CTCF proximal LADs with changes and the number of non-CTCF proximal LADs with changes. Based on the DamID traces shown I would guess these percentages to be very small so that the very minor shifts in the global curves likely reflect larger changes at very few loci rather than a cumulative minor shift and tests should be done on the data and more traces added that can address this point.

[See reviewer 1 for a similar comment.]

We now include an analysis of the local gain outside LAD borders (new Fig. 1F), which suggests a small global increase rather than a strong increase at several LAD borders. We have also tried to substantiate our statements on LAD border positioning by including actual numbers in the text (l. 199-200, 252-257).

The one more striking shift observed in panel E is a gain in nuclear lamina association in the adjacent inter-LAD region upon CTCF depletion. This is interpreted as meaning that CTCF contributes to the sharpening of LAD borders, but there are many other possible interpretations of the data, including mechanical ones. It could be simply that the CTCF-based loops help hold the DNA more internally in the nucleus so that it is harder to establish lamina interactions with a larger region that has affinity or many other possibiltiies including those involving epigenetics. Thus, such conclusions that seem to be trying to get the reader to just accept their particular interpretation need to be removed from the text unless additional data is shown that discounts the other hypotheses.

With “a sharpening of LAD borders”, we tried to describe the observed difference in LaminB1 traces. We did not intend to provide an interpretation of the data. We have rephrased the text to better reflect this (l. 211-215).

They next test the role of cohesin by its indirect stabilization or depletion with AID- WAPL and AID-RAD21. Again there is the issue that it is unclear what percentage of 7 the different types of LAD borders are affected and this should be added, but there were also some slightly larger changes in the global profiles. Nonetheless, there were also more significant changes in the body of the LADs themselves which suggests that just as depletion of lamins can yield massive changes in the nucleus to generate lots of indirect artefacts, it is also quite possible that altering cohesin even in the short term would have similar indirect effects. Despite the textual statements that cohesin is playing a clear role, to me the traces of the combined CTCF and WAPL depletion look extremely similar to that of CTCF alone back in Figure 1. I would really like to see these traces overlaid as to me this would suggest that the predominant effect is through CTCF depletion and that the effects seen when affecting cohesin alone are likely indirect.

We have included more numbers of the amount of LAD borders (l. 199-200, 252-254) and LADs (l. 329-331) that are affected.

We concur that the depletion of CTCF and double depletion of CTCF/WAPL have very similar effects on the focal detachment of CTCF sites, while WAPL depletion (and thus stabilization of cohesin on the DNA) by itself strongly induces this detachment. This is why we propose a model where CTCF and cohesin together are required for focal detachment at LAD borders and within LADs.

The narrative continues with the assumption that CTCF binding actively drives the detachment of a never clearly stated amount of inter-LADs chromatin as a directed function and next I was a little confused with the terminology describing the CTCF sites being inside LADs: since these sites are detached from the nuclear lamina then since the definition of a LAD is being at the lamina I don't think they should be called as part of LADs. Do the authors mean facultative LADs that are changing? or that they are only weak breaks from the LAD in traces because only a small subset of the pooled population has them broken away from the nuclear lamina? ...

Indeed, the detachments are very local and relatively weak (i.e, involving only a subset of cells in the pool), and therefore the algorithm that we routinely use to call LADs does not score them as interLADs. We now mention this in the text (l. 279- 281).

... Regardless, it was also unclear whether the actively transcribed genes that are broken away also from LADs also have CTCF, but they show no effect of depletion on the actively transcribed gene regions broken away from LADs whereas the CTCF islands that are not actively transcribed genes in regions that have broken away from LADs were affected. This all needs to be cleared up and described better, but, again, from the standpoint of overintepreting, I would think it equally likely that the CTCF and transcription likely prevent the spread of epigenetic marks through these regions and/or sterically block attachment to the lamina and when the CTCF goes away this no longer happens and it gets marked and bound. So to address this properly would be to NOT drop further study of the transcriptionally active sites as they did, but rather test what happens when they knock off the transcription factors and block transcription and see if that then results in those regions associating with the lamina +/- co-depletion of CTCF - that is if the CTCF is actually also in those sites. Then the other thing would be to do the ChIP-Seq for the epigenetic marks and see if they change with the CTCF depletion in the non-transcriptionally active 8 CTCF islands. Note also that I was also not clear in this section on whether the epigenetic data were all previous data? I assume so because there is no distinction about changes due to CTCF depletion and no epigenetic ChIP-Seq is described in the methods; however, it does seem like it might have been interesting to check if these change with the CTCF and other depletions generally for other experiments as well and it seems also that not doing so implies that these are not changing when they may well be.

The epigenetic data are indeed from previous publications. This is a common practice in the field, and we feel that this is justified because it would be an enormous effort to generate all these data ourselves. However, we now include new ChIP-seq data for H3K27me3 +/- CTCF depletion (new Fig. 6A). This is the only mark that we find specifically enriched at CTCF sites, and hence this was worthy further investigation. We believe that the other proposed experiments (“knock off the transcription factors and block transcription”) are too much outside the scope of this manuscript, which focuses on the role of CTCF and cohesin. Effects of transcription have been studied in depth elsewhere (e.g. Therizols et al, Science 2014; Brueckner et al, EMBO J 2020).

Re the need for new data, it was not clear what data was being used to calculate loop anchors, but this seems too like something where to do it properly one should get Hi-C data from the same +/- CTCF system used. In general, it was possible to find some answers to questions about what data was used by sifting through the methods section, but I think this should be being stated in the text throughout what data was used and likewise what external datasets - note this latter is relegated entirely to the supplementary information, but this is information that belongs in the results section. Sifting through the methods also explained with how LADs were called why these non-LAD regions are being called as within LADs, but I still think it would be more clear to reduce the bin sizes and to not call them LADs in the first place. So there needs throughout the text to be more orientation of the reader as to what data is actually being used and what is being compared so that they do not have to keep checking the supplementary data, figure legends, and methods to still sometimes only guess at this.

All the external data used is already referenced in the figure legends and/or results section. We have clarified this to prevent any confusion (e.g. l. 1046). The supplementary information provides the complete list of external data with data identifiers.

The reviewer specifically points out that we should use Hi-C data from the same CTCF-AID model used to determine loop anchors. However, we think that this is an unreasonable request. A complete and reliable list of loop anchors can only be determined from a very deeply sequenced Hi-C library, which is very expensive and unnecessary.

Finally, the reviewer suggests to reduce the bin size to call locally-detached features (active genes, CTCF binding sites) as separate iLAD regions. However, we want to point out that these features are still enriched at the nuclear lamina with z-scores > 0. Smaller bin sizes will thus not result in a transition from LAD to iLAD. Furthermore, we previously observed that small genomic bins result in significantly more noise (van Schaik et al, EMBO Rep 2020). 10 kb bins provide the best balance between data resolution and data quality.

The correlations in Figure 4 strike me as not being as great as the text would imply. I can understand why Spearman and not Pearson was used here, but even for Spearman to my understanding a strong correlation is at 1 and their plot seems to only go to about 0.25 according to their gradient key. Yet they state that there are significant correlations. What is potentially interesting is the way some parameters switch from leaning towards correlating to anticorrelating between different depletions, but presented as it is this data does not give the confidence of interpretation that the text does. Perhaps their color system is not as good as just putting the numbers in the individual boxes and it would be better to remove the color gradient and just shade the boxes with significance and then put the actual numbers inside, but from how the data is presented I am hesitant to embrace the textual interpretations here. At the same time, if reasonable numbers were clearly given then I would say this is a place where there are some more interesting relationships where we could say the depletion more affects a particular chromosome region etc. At the same time, this data also needs to be elaborated and expanded on if this is the case because for example for the telomere suggested correlation one needs to also see the distribution of both LADs and CTCF binding sites at telomeres to judge whether this might just be an effect due to greater densities of CTCF binding here or something reflecting a unique function here. This would also require knowing what percentage of sites in these regions changed compared to other regions controlled for density and other factors. Also at the same time, a correlation like the small LAD with CTCF versus the big LADs with WAPL could just reflect the possible general genome-wide pleiotropic effects of messing with cohesin for why larger LADs tend to be affected and not necessarily something reflecting a specific functionality.

We thank the reviewer for the good suggestion to add the actual correlations in the figure, we have changed the figure accordingly. We have also included a supplementary figure with the correlations between the LAD features (Fig. S8). This illustrates that CTCF density and telomere proximity are only weakly correlated (Spearman: -0.22), which is not enough to explain the observed correlations in Fig. 4.

The reviewer also indicates that our observed Spearman correlations are not sufficient to support our interpretations in the text. However, given that our LAD features are an obvious oversimplification of LAD domains and that we find complex patterns with multiple significant features, we would argue that the observed correlations (up to 0.38) are very meaningful. Correlations near 1 are rarely found in genome-wide analyses of spatial organization and epigenetics.

Next the transcription Figure 5 is straigtforward but just negative data and the epigenetics Figure 6 would have been better to do ChIP-Seq +/- the depletions than using inhibitors. Moreover, there could easily be combinatorial epigenetic signatures that would only be identified by looking at multiple marks in the various depletion lines. Thus, to conclude that there is no interplay with even just the one is perhaps premature, but the implication would appear to be that epigenetics in general is not likely interplaying and since more was not tested there should be a lot more qualification of these conclusions. This gets to the biggest issue, I highlighted a number of individual points above where it would be worth doing a bit more analysis of the existing datasets and also perhaps do a bit of ChIP-Seq on the lines generated for this study, but with the data that hs been presented there really needs to be a significant cut back in the strength of conclusions made throughout. In particular, the conclusions as stated in the abstract are that CTCF is significantly at LAD borders, but its depletion doesn't change much, yet nonetheless it is important for "reinforcing" LAD borders and is important to "mediate fine-tuning of NL interactions". The data is clear that some fraction of LADs change when CTCF or cohesin are depleted, but whether the function is to reinforce is never actually tested in any meaningful way and the use of the term fine-tuning is also not supported with any actual data. Just that some small fraction has an effect does not mean the role is to fine tune something. What are the specifically so-regulated genes and functional consequences of their role if they are really fine tuning? If one was looking at a functional output such as transcription, then this term would suggest that the transcription factors etc are around but some aspect of the CTCF interaction with the LAD gives an additional bit of regulation to the system such as making the on/off regulation of the gene tighter or that normally CTCF goes off at a certain time to affect the temporal dynamics of the gene's regulation with a particular defect that is apparent that could lead to pathology if it did not have this extra bit of fine-tuning regulation. However, no experiments show anything like this for any kind of functional output and I am not certain what would be useful to test here since they dropped investigating transcription early on. So the conclusions strike me as massively overstated.

The reviewer again suggests to profile multiple epigenetic marks during the protein depletion experiments. However, besides the ATAC-seq data that we already generated, we are unsure which marks this should be. The only factor that is specifically enriched at CTCF binding sites is H3K27me3. We have therefore extended the analysis in Fig. 6 with ChIP-seq data for H3K27me3 +/- CTCF depletion (l. 401-404). Together with the inhibitor data, we believe that this analysis provides strong evidence that there is no interplay between CTCF and H3K27me3 to maintain LAD borders.

Next, the reviewer points out that our data does not support our conclusions, but we believe that this mostly relates to misunderstandings. For example, we did not intend to suggest that fine-tuning of LAD borders by CTCF affects the function of the LAD border. Rather, we tried to describe that CTCF binding results in a more consistent placement of LAD borders. We have tried to change the phrasing of our conclusions to prevent this confusion (l. 51-52, 211-215, 490-491).

Another general question I have is where did the 20kb cutoff come from and how valid is it? It was stated that the peak was at 10kb, but with the accuracy of mapping LAD boundaries this could be even closer and even 10kb could be physically far from the nuclear envelope. We still don't know in what form DNA interacts with the nuclear lamina, but at 200 bp per nucleosome and 10-11 nm wide in a 10 nm fiber, even without linker histones at lower density, this would be 100 x 10 nm = 1 μm which is 1/5 the diameter of the average mammalian nucleus. Even if assuming that 30 nm fibers are interacting and using one of the tightest packing models at a 10x further shortening, this would still be far enough away that it is hard to imagine CTCF binding sites at this distance having a huge impact on the specific binding of the LAD boundaries. So just changing these parameters could potentially find correlations that were not apparent with the current analysis. It also would be beneficial to know numbers for what percentage of a particular subgroup of CTCF-associated LADs (versus non-associated) does something throughout the study. For example, what percentage of the LADs in each category are facultative vs constitutive and how many of the facultative are enriched for developmentally regulated genes. We get in Supplemental Figure S5 a statement that CTCF depletion and cohesin perturbation affect overlapping sets of differentiation genes, but nowhere is this data presented in a way that give the reader a sense of whether this is 90% of the genes changing in differentiation having some CTCF/cohesin connection or regulation or if it is 5% of the genes changing in differentiation or if CTCF/cohesin is enriched at developmentally changing genes since there could be an extra player in the regulation at this particular gene subset. It seems that after spending so much time and effort in making these elegant depletion systems and generating so much data looking at LAD changes that they should be asking these questions with the data.

The reviewer first questions our cut-off of 20 kb for CTCF binding sites near LAD borders. In several cell lines, we consistently observe that CTCF binding sites are enriched within 20 kb outside of LAD borders. This distance is similar to previous reports (Guelen et al, Nature 2008; Harr et al, J Cell Biol 2015). To understand whether this enrichment has a functional consequence for LAD border positioning, we therefore based our segmentation on this distance. We do appreciate that 20 kb translates to a potentially long DNA fiber, which is fascinating and one of the puzzles to be solved in the future.

Next, the reviewer asks for more numbers to support our statements. We have added figures that quantify the amount of LAD (borders) affected by the protein depletions (i.e. Fig. 1F, 4B) and the overlap in differentially expressed genes between protein depletions and the differentiation data (Fig. 5B, S7C). However, to prevent a data overload and keep the manuscript readable, we don’t think we should include analyses, figures and numbers for all possible comparisons

**Reviewer 3**

CTCF and cohesin are major players in A-compartment genome organization and it has been long known that CTCF is enriched at LAD borders and, more recently, in local detached euchromatic regions within LADs. What has remained uncertain is the role that CTCF plays in the organization of LADs, if any. In this manuscript, van Schaik et al describe a series of acute depletion experiments to determine how CTCF and cohesin regulate LAD organization and borders. In addition, van Schaik et al investigate the role that H3K27me3 plays in CTCF regulated genome interactions and in LAD contacts with the nuclear lamina. Overall this is an intriguing set of studies that helps to clarify and demonstrate the LAD organization is independent of A-compartment organizing principles. There are some concerns with experimental set-up and analyses (outlined below) which dampen enthusiasm for this work as-is a little. Nonetheless, these studies are a valuable addition to the field and a necessary step forward in our understanding of LAD organization.

We thank the reviewer for the helpful and positive comments.

1. A general comment that applies to all the experiments outlined: given the perturbations, there should be an analysis of the cell cycle status and cell doubling during experimental manipulation time-frames. If the cycle is slowed or halted, in what cell cycle stage? If the cells arrest in G1/S/G2, this could mean that the perturbations do not affect the organization of established LADS, but would say nothing about whether reorganization requires these factors/chromatin modification (in the case of H3K27me3).

We agree that this is an important control that was missing. We have now included a comprehensive analysis of cell cycle stages after auxin-mediated protein depletion of CTCF, RAD21 and CTCF/WAPL in Fig. S3 (l. 189-191, 230-233). A similar analysis has been performed after WAPL depletion (Liu et al, Nature Genetics 2021). RAD21 depletion strongly affects the cell cycle, but the other depletions have no or only minor effects and thus cannot explain the observed differences in LAD organization

.

2. Data on LADs with active genes within 10 kb or the LAD border were excluded from the analyses. I am puzzled by this. The authors state this was to avoid "confounding factors", but this might be important to understanding LAD organization. I would like to see the parsed out data in a supplemental figure. Does it deviate from the filtered LAD border data? If so, how/why? It is unclear to me what confounding factors could precludes this inclusion or at least separate analyses in supplemental data. The two features are not unrelated, after all.

It is well established that active transcription induces detachment from the nuclear lamina. We therefore expected that LAD borders with active genes would be stable irrespective of the presence of CTCF. We have clarified this in the main text (l. 146). However, we do appreciate that CTCF and transcription are not unrelated and agree that a separate analyses of LAD borders with both features should be included to provide a complete overview. We have now included this analysis in Fig. S4D- F (l. 204-207).

3. Looking at the western blots for the CTCF-AID, WAPL-AID, RAD21-AID and for both CTCF and WAPL-AID, it appears that CTCF and WAPL levels are *already* compromised prior to auxin mediated degradation. This could mean that the cells are already compromised. All data should have the parental control (not just 0 hours) as a baseline control. One concern is that the cells have compensated (in some way) for a depleted level of these factors thus obscuring some potentially important roles. [See reviewer 2 for a similar comment.]

We agree that compromised protein levels as a result of AID-tagging are a potential limitation of this study (and many others), which should be described very clearly. We now emphasize better that CTCF and WAPL are already partially depleted prior to auxin-mediated depletion (l. 169, 228). To determine the consequence of the AID-mediated depletion, we now compare the CTCF detachment between the untreated time point (0 h) and the parental clone (with normal protein levels) in Fig. S6A (l. 289-300). Although the biggest effect is observed upon depletion of the remaining protein (see Fig. 3C), we do observe that the initial protein depletion of CTCF and WAPL also has an effect. This confirms a dose-dependent effect.

Note that we generated new PT data that includes a time point of IAA treatment (24 h) to determine the effect of IAA itself on the cells and to better estimate the expected variation at borders / LADs (see point 5). However, these new data were generated with a new batch of antibody against LaminB1 and seem of superior quality. We use this new data throughout the manuscript, except for the analysis in Fig. 3C / S6A where the old data (which lacks an IAA time point) better matches the other data. This difference in data quality does not affect our conclusions because all remaining comparisons are performed between time points within clones.

4. "Furthermore, the effects of RAD21 and WAPL on LAD-wide NL interactions both depend on the chromosomal position (i.e., distance to telomere and centromere), but in opposite orientation. This effect appears roughly linear with distance to the telomeres and is present across all chromosomes (Fig 4E). Chromatin nearby telomeres is enriched at the NL in early G1 cells and slowly loses interactions with the NL in interphase [33]. Possibly stabilized cohesin somehow prevents this normal maturation of NL interactions" This once again highlights the need for a robust cell cycle analysis of the perturbed cells. This could be reflecting alterations in the cell cycle itself.

As written in response to the first comment, we have now included a cell cycle analysis. While RAD21 is highly essential and already after 6 hours strongly perturbs the cell cycle distribution, CTCF and WAPL depletions have no or minor effects on the cell cycle. This analysis thus rules out the possibility that the observed correlations are solely reflecting alterations in the cell cycle.

5. "Towards this goal we calculated mean pA-DamID scores on a consensus set of LADs across all conditions, and then determined the changes in scores in each depletion experiment (see Methods, Fig S4)."

I am concerned about the validity of this approach given that pA-DamID (DamID generally) is not known a quantitative measure of degree of association (likely there is some, but this has not been calibrated to be so). In order to discern the validity of this approach, parental cells need to be evaluated the same way (with each other between replicates and against the amalgam LAD maps). This is also a concern since the degree of depletion in the untreated cells varies (especially since some are starting with lower protein levels to begin with in the genetically engineered lines).

DamID scores are highly correlated to radial positioning of genomic loci {e.g. Guelen et al, Nature 2008; Peric-Hupkes et al, Mol Cell 2010} and single-cell contact frequencies with the nuclear lamina (Kind et al, Cell 2015), which underlines that DamID is a quantitative measure. Given the strong concordance between DamID and pA-DamID data (van Schaik et al, EMBO Rep 2020), it is likely that the same applies to pA-DamID data as well.

We do, however, agree that a similar IAA time course in PT cells is an important control that was missing. We have now included this (i.e. Fig. 1E, 4A, S6B). These data illustrate that the observed differences are not caused by IAA itself and that the observed differences after CTCF, RAD21 and WAPL depletions are larger than expected.

6. In the discussion regarding H3K27me3, it is suggested that H3K9me2 might be sufficient for LAD targeting, but this contradicts the findings that H3K9me2 alone is insufficient -as evidenced by H3K9me2 enriched chromocenters in mouse cells. Perhaps some of the findings can be explained by redundancies in organization? Also, the role of active transcription—either at LAD borders or generally may paly a role in this process.

We did not intend to suggest that H3K9me2 is the only player (we did not use the word "sufficient"). We have slightly reworded the passage (l. 498-500).

7. The overall discussion of H3k27me3 in LAD organization is excellent, however given the overlap of CTCF with H3K27me3 and the role that LADs/H3k27me3 play in facultative gene regulation, is it possible that in ESC some of these findings are due to bi-valent or semi-poised genes? I think further discussion of the nature of gene regulation in ESC versus other cell types is warranted. In addition, the make-up of the lamin proteins and the nuclear envelope varies between cell types, notably the lamins themselves. (ESC having no lamin A, but instead reliant on LBR—Solovei 2013).

We have now emphasized that our findings may not necessarily extrapolate to other cell types (Discussion, l. 425-428). There are indeed many differences in chromatin and NL makeup between ESCs and other cell types, but because none of our data directly relate to this, we feel that an extensive discussion of such differences would distract from the main messages of our manuscript. Please note that, in response to a comment by reviewer #2, we have now included additional ChIP-seq data of H3K27me3 after depletion of CTCF (new Fig. 6A).

**2^nd^ round**

**Reviewer 1**

I have carefully reviewed comments from the reviewers and corresponding responses from the authors. The authors have significantly improved the manuscript based on the reviewer comments. Generating new Hi-C data from the same CTCF-AID model used to determine loop anchors, as suggested by reviewer 2, would be useful. However, I agree with the authors that this is not strictly necessary for the paper. The same goes for the general request to generate more ChIP-seq data (beyond the new H3K27me3 data generated).

I suggest accepting the paper.
